# Computational drugs repositioning identifies inhibitors of oncogenic PI3K/AKT/P70S6K-dependent pathways among FDA-approved compounds

**DOI:** 10.18632/oncotarget.11318

**Published:** 2016-08-16

**Authors:** Diego Carrella, Isabella Manni, Barbara Tumaini, Rosanna Dattilo, Federica Papaccio, Margherita Mutarelli, Francesco Sirci, Carla A. Amoreo, Marcella Mottolese, Manuela Iezzi, Laura Ciolli, Valentina Aria, Roberta Bosotti, Antonella Isacchi, Fabrizio Loreni, Alberto Bardelli, Vittorio E. Avvedimento, Diego di Bernardo, Luca Cardone

**Affiliations:** ^1^ Telethon Institute of Genetics and Medicine, Pozzuoli, Naples, Italy; ^2^ Department of Hematology, Oncology and Molecular Medicine, Biobank Unit, Istituto Superiore di Sanità, Rome, Italy; ^3^ Department of Research, Advanced Diagnostics, and Technological Innovations, Regina Elena National Cancer Institute, Rome, Italy; ^4^ S.C. Anatomia Patologica, Regina Elena National Cancer Institute, Rome, Italy; ^5^ Immuno-Oncology, Laboratory, Aging Research Center, G. d'Annunzio University of Chieti, Pescara, Italy; ^6^ Department of Biology, University of Rome Tor Vergata, Rome, Italy; ^7^ Nerviano Medical Sciences SRL, Nerviano, Italy; ^8^ Candiolo Cancer Institute-FPO, IRCCS, Candiolo, Torino, Italy; ^9^ Department of Oncology, University of Torino, Candiolo, Torino, Italy; ^10^ Dipartimento di Medicina Molecolare e Biotecnologie Mediche, Università degli Studi di Napoli “Federico II”, Naples, Italy; ^11^ Department of Electrical Engineering and Information Technology, University of Naples “Federico II”, Naples, Italy

**Keywords:** oncogenes, PI3K-dependent pathways, gene expression signatures, drugs network, FDA-approved drugs

## Abstract

The discovery of inhibitors for oncogenic signalling pathways remains a key focus in modern oncology, based on personalized and targeted therapeutics. Computational drug repurposing via the analysis of FDA-approved drug network is becoming a very effective approach to identify therapeutic opportunities in cancer and other human diseases. Given that gene expression signatures can be associated with specific oncogenic mutations, we tested whether a “reverse” oncogene-specific signature might assist in the computational repositioning of inhibitors of oncogenic pathways. As a proof of principle, we focused on oncogenic PI3K-dependent signalling, a molecular pathway frequently driving cancer progression as well as raising resistance to anticancer-targeted therapies. We show that implementation of “reverse” oncogenic PI3K-dependent transcriptional signatures combined with interrogation of drug networks identified inhibitors of PI3K-dependent signalling among FDA-approved compounds. This led to repositioning of Niclosamide (Niclo) and Pyrvinium Pamoate (PP), two anthelmintic drugs, as inhibitors of oncogenic PI3K-dependent signalling. Niclo inhibited phosphorylation of P70S6K, while PP inhibited phosphorylation of AKT and P70S6K, which are downstream targets of PI3K. Anthelmintics inhibited oncogenic PI3K-dependent gene expression and showed a cytostatic effect *in vitro* and in mouse mammary gland. Lastly, PP inhibited the growth of breast cancer cells harbouring PI3K mutations. Our data indicate that drug repositioning by network analysis of oncogene-specific transcriptional signatures is an efficient strategy for identifying oncogenic pathway inhibitors among FDA-approved compounds. We propose that PP and Niclo should be further investigated as potential therapeutics for the treatment of tumors or diseases carrying the constitutive activation of the PI3K/P70S6K signalling axis.

## INTRODUCTION

Targeted inhibition of oncogenic signalling pathways represents the main goal of modern oncology: selective inhibitors of oncogenes such as mutated kinases have been identified mainly through traditional small molecule drug screening, aimed at identifying inhibitors of their catalytic activity or cellular localization. However, ever-increasing failure rates, high cost, unsatisfactory safety profile, and limited efficacy are often associated with such traditional drug screens. Moreover, these inhibitors, even when effective, show paradigms of primary or secondary resistance [1], [2], highlighting the need for novel efficacious and cost-effective strategies to identify efficacious modulators of oncogenic pathways in cells. Gene signatures associated with oncogenic activation of molecular pathways may offer novel opportunities for targeted therapeutics discovery in cancer. Specific gene expression signatures can be associated with oncogenic mutations and deregulated signalling pathways in tumors [3], [4], [5]. One opportunity to implement these molecular data into drug discovery is offered by the inspection of drug networks. These networks can be derived by computing the similarity between gene signatures generated by drug treatments in specific cell lines, with the assumption that each drug treatment generates a drug-specific signature [6]. In this drug-network, drugs can be grouped into communities composed of drugs that produce similar transcriptional profiles and hence may act through a similar Mode of Action (MoA) [7]. This network-based analysis allows to identify the MoA of novel compounds, as well as, to repurpose drugs for novel therapeutic indications (“drug repositioning”) [7], [8]. Moreover, the inspection of drug networks with disease- or oncogene-associated signatures might help repositioning drugs able to “revert” a disease signature and, thus, the disease phenotype [9].

The PI3K proto-oncogene and its downstream pathways offer a relevant paradigm of targeted therapies in oncology. The PI3K/AKT/mTOR/-dependent pathways include key modulators of cell proliferation, survival and metabolism in epithelial cells [10], [11]. The aberrant activation of these pathways has been identified as the determinant driver event of tumorigenesis and constitutes an important factor in the anticancer drug response and clinical prognosis in different tumor types [12], [13], [14]. In epithelial cancer, the PI3K-dependent signalling pathway is most frequently activated by genetic alteration such as gene copy number variation or somatic mutations in *PIK3CA*, the gene encoding the p110α catalytic subunit of PI3K or inactivating mutations in PTEN [15], [16]. Three recurrent oncogenic “hotspots” account for the majority of somatic *PIK3CA* mutations. Two of these mutations, E542K and E545K, occur in the helical domain, and the third mutation, H1047R, affects the kinase domain. All three mutations result in enhanced lipid kinase activity and activation of downstream targets such as the AKT, P70S6K and S6 proteins. Pharmacological strategies aimed at inhibiting the oncogenic activation of the PI3K-dependent pathways are under active pre-clinical and clinical investigation [17], [18], [19]. However, paradigms of tumor resistance after PI3K/mTOR inhibitor treatments are emerging [20], [21], highlighting the need for alternative approaches to inhibit this pathway.

To test whether an oncogene specific gene signature might assist in computational repositioning of selective inhibitors of oncogenic pathways, we used the PI3K oncogene as a test case. The approach is based on the following hypothesis: if the gene signature summarizing the effect of a drug is “anti-similar” with an oncogenic pathway-derived signature, it is reasonable to expect that this drug acts as pathway inhibitor, able to revert the oncogenic signature. To this end, we queried a drug network with an anti-similar (or reverse) oncogenic PI3K-dependent gene signature derived from somatic knock-in cellular models. Computational analysis effectively identified well-known selective inhibitors of PI3K-dependent signalling among FDA-approved compounds. Moreover, we repositioned Niclosamide (Niclo) and Pyrvinium Pamoate (PP), two anthelmintic drugs, as effective inhibitors of oncogenic PI3K-dependent signalling by inhibiting the activation of the AKT/P70S6K signalling axis.

## RESULTS

### Gene expression-based drug network analysis repositioned anthelminthic drugs as potential inhibitors of oncogenic PI3K-dependent pathways

**Figure 1:**
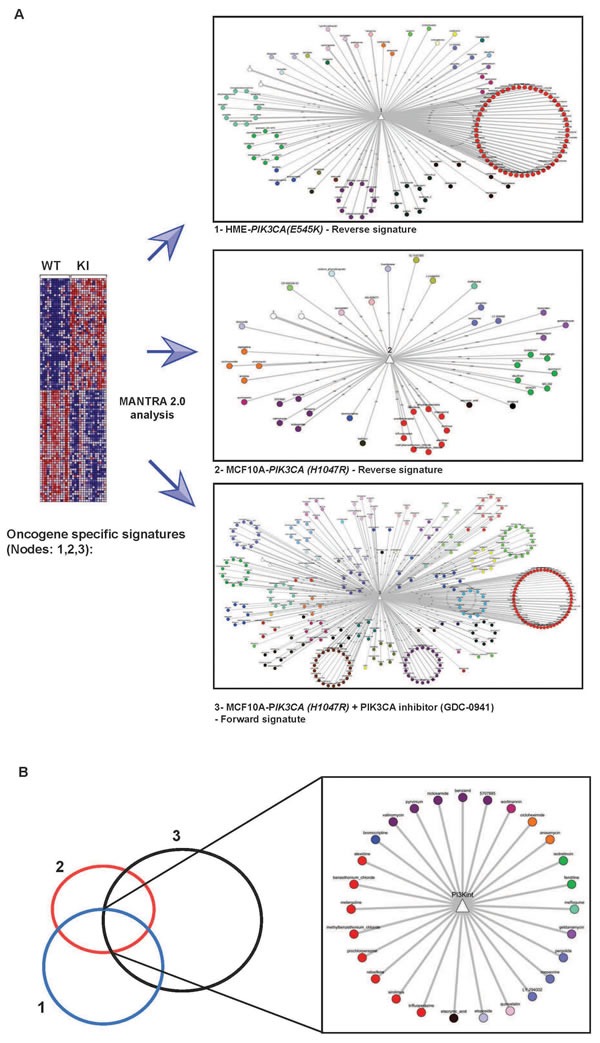
Computational drug repositioning identifies possible inhibitors of the PI3K-dependent pathway **A.** Schematic representation of the drug repositioning approach used in this study. Reverse (1 & 2) and forward (3) oncogenic PI3K-specific gene expression signatures derived from isogenic cell lines generated new nodes in MANTRA network (See text and Experimental Procedures). Drugs are organized in a network of nodes (drugs) and edges (similarities) highlighting “communities” of drugs sharing a similar MoA. Edges display drugs with significant computed distance. Different colors refer to different drug communities. **B.** Drugs common to networks 1, 2 and 3 were selected and a new node was identified, here referred to as a PI3K intersection (PI3K int).

We have previously developed a computational approach to predict drug MoA and drug repurposing by using the analysis of the Connectivity Map (Cmap) [22], a compendium of gene expression profiles (GEPs) following drug treatment of human cell lines with 1,309 bioactive small molecules. The approach was based on generating a single “prototype” ranked list (PRL) of differentially expressed genes for each drug following treatment across multiple cell lines, or at different dosages [7, 23]. We have developed a new version, online community-based resource (referred as MANTRA 2.0) that supports this process by exploiting similarities between drug-induced and disease-induced transcriptional profiles [24]. To reposition FDA-approved drugs that might act as inhibitors of oncogenic PI3K­-dependent pathways, we queried the MANTRA 2.0 drug network. To generate oncogenic PI3K-dependent gene signatures, we took advantage of isogenic Knock-In (KI) cell lines, in which a normal allele in non-transformed human mammary epithelial cells had been replaced with *PIK3CA(E545K)* or *PIK3CA(H1047R)* alleles by somatic adenovirus-mediated recombination [25-27]. Since these cells essentially differ from their isogenic wild type counterpart in the expression of mutated PIK3CA protein only, they allow for the generation of a specific bona-fide oncogenic PI3K-dependent gene signature. From each signature, we generated a PIK3CA-reverse signature by sorting genes in reverse order of differential expression (i.e. the most down-regulated ranked at the top of the signature, while the most up-regulated at the bottom) to generate a gene expression profile associated with inhibition of oncogenic PI3K-dependent pathways (Figure 1A and M&M). We also used a gene expression signature obtained from a MCF10A cell line carrying the *PIK3CA(H1047R)* mutation treated with GDC-941, a selective inhibitor of the catalytic subunit of PI3K. Thus, we generated three new nodes in the MANTRA network here referred to as: 1) *PIK3CA(E545K)*-reverse; 2) *PIK3CA(H1047R)*-reverse and 3) *PIK3CA (H1047R)* plus inhibitor (Figure 1A). Computational distance analysis of these transcriptional signatures in the MANTRA 2.0 network allowed us to identify drugs and communities with significant distance (threshold ≤0.86) from each of the PIK3CA-specific nodes (Figure 1A and Table S1). Compounds common to all three networks were then selected (Figure 1B and Table S1). This produced a list of 27 compounds (Table 1) that are predicted to generate transcriptional signatures with significant similarity to the ones generated by the inhibition of oncogenic PI3K signalling. In support of the predictive power of our approach, we identified LY-294002 and Wortmannin, two well-known inhibitors of p110α catalytic subunit of PI3K. We also identified Sirolimus (rapamycin), a clinically relevant small molecule inhibitor of mTOR pathway and Quinostatin, an inhibitor of the p110α catalitic subunit of PI3K [28] (Table 1). Drug-network analysis also allows us to identify enrichment of drug communities from each of the *PIK3CA*-specific nodes (Table S2), indicating drug classes significantly close to each node. Inspection of communiti enrichment common to all three nodes strongly suggested that antipsychotics, sodium/calcium decreasers, calcium channel blockers and Na+/K+-ATPase (sodium potassium) membrane pump inhibitors are predicted to negatively regulate PI3K-dependent signalling *in-silico*. On the other hand, data in Table S2 also indicate that these drug classes might inhibit PI3K-dependent signalling as a potential off-target effect. Notably, the repositioning approaches based on “reverse” genetic signatures (networks 1+2) was sufficient to identify 6 out of top 10 communities identified through the use of a specific inhibitor-derived signature, thus indicating that the “reverse” signature-based strategy could help in repositioning studies for which specific pathway inhibitors are not yet available.

Among selected drugs (Table 1), we focused our attention on Niclosamide (Niclo) and Pyrvinium Pamoate (PP), two FDA-approved anthelmintic drugs, as a result of following considerations: i) recent preclinical data have outlined anticancer effects of both compounds in several *in vitro* and *in vivo* cancer models [29-33]; ii) the possibility that these compounds can inhibit the oncogenic activation of PI3K-dependent pathways was never directly tested. We thus decided to experimentally validate the *in silico* repositioning of PP or Niclo acting as inhibitors of oncogenic, constitutively active, PI3K-dependent signalling.

**Table 1:** Rank-ordered list of compounds after MANTRA 2.0 query and post-processed selection

Drug name	Community	Therapeutic Indications	Drug Class
5707885	62		-
Alexidine	100	Bacterial Infections	-
Anisomycin	53	Infection Mycoses	Protein synthesis inhibitors
Benzamil	62		Sodium/Calcium Decreasers
Benzethonium_chloride	100		-
Bromocriptine	73	Parkinson Disease	Dopamine receptors agonists
Cicloheximide	53		Protein synthesis inhibitors
Etacrynic_acid	104		-
Etoposide	3	Neoplasms	G1/S Cell Cycle Blockers
Fendiline	40		Calcium signal modulators
Geldanamycin	28	Gram-Negative Bacterial Infections	HSP9- inhibitors
Isotretinoin	40	Acne Vulgaris Skin Diseases	-
LY-294002	16		PI3K inhibitors
Mefloquine	34	Malaria	Antihistamines
Mepacrine	16	Helminthiasis	-
Metergoline	100	Fever Pain	Antipsychotics
Methylbenzethonium_chloride	100		-
Niclosamide	62	Helminthiasis	Antiinfectives, Antiseptics, Antiparasitics
Pergolide	16	Parkinson Disease	-
Prochlorperazine	100	Neoplasms Vomiting	Antipsychotics (Phenothiazines)
Pyrvinium	62	Helminthiasis	Antiinfectives, Antiseptics, Antiparasitics
Quinostatin	4		PI3Ks inhibitors
Raloxifene	100	Osteoporosis	calcium channel blockers and Ca2+ level increaser
Sirolimus	100		-
Trifluoperazine	100	Vomiting	Antipsychotics(Phenothiazine)
Valinomycin	62	Neoplasms	-
Wortmannin	60		PI3Ks inhibitors

Nodes scoring higher than 0,86 were considered not significant. Note that some identified compounds are well known pharmacological inhibitors of PI3K signalling, such as LY-294002, Wortamannin and Quinostatin. Sirolimus, a rapalog drug targeting downstream PI3K-signalling, was also identified. The table indicated the drug class, therapeutic indication and the community number of each drug according to the MANTRA database.

### Niclo and PP control oncogenic PIK3CA-dependent gene expression

**Figure 2:**
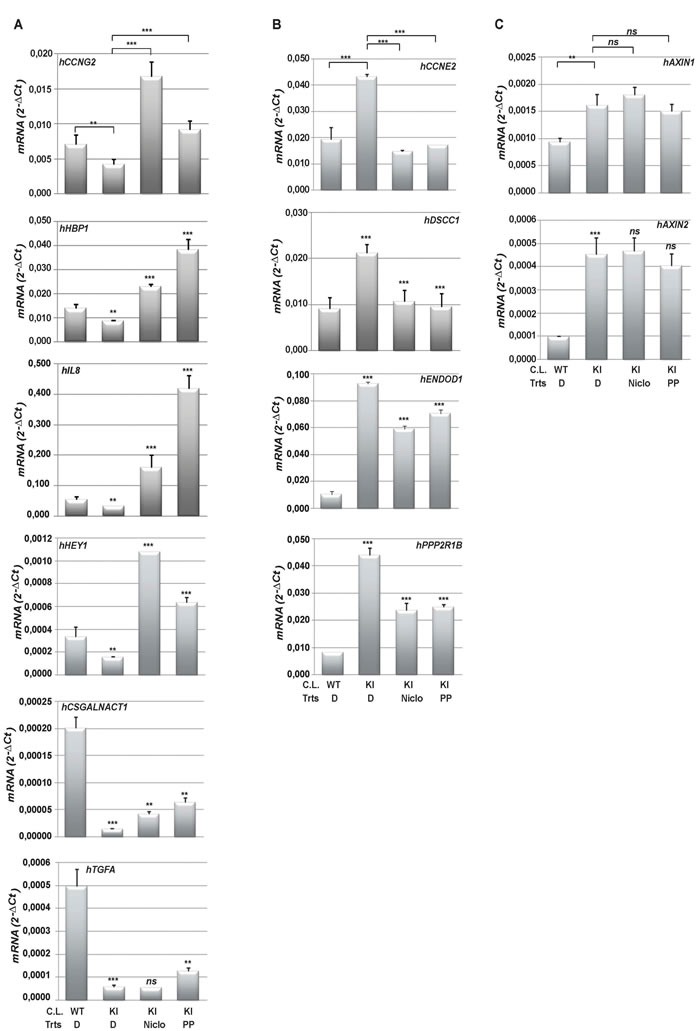
Niclo and PP effectively control oncogenic PI3K-dependent gene expression **A.** and **B.** Total RNA was harvested from wild type HME(WT) or an isogenic clone carrying a *PIK3CA(E545K)* mutation (KI) after treatment (Trts) with vehicle (DMSO (D)), Niclo or PP. The most differentially expressed genes were predicted and selected as described in M&M. The expression of genes down-regulated by oncogenic PIK3CA (*hCCNG2*, *hHBP1, hIL8*, *hHEY1, hTGFA and hCSGALNACT1)* **A.** or up- regulated by oncogenic PIK3CA (*hCCNE2*, *hDSCC1*, *hENDOD1* and *hPPP2R1B)* **B.** and selected WNT target genes (hAXIN1 and hAXIN2) **C.** was analyzed by quantitative real time PCR. Data indicate absolute values ± SD and are the average of at least three independent experiments. Statistical significance was evaluated through *t-*test, performed for the following conditions: DMSO-treated WT Vs DMSO-treated KI or DMSO-treated KI Vs Drugs-treated KI. **p < 0.05, ***p < 0.01, ns: not significant. C.L.: Cell Line.

In order to confirm the effective regulation of gene expression by Niclo and PP and their ability to “reverse” the oncogenic PI3K-dependent gene expression signature, we selected, among the most up-regulated and down-regulated genes from the *PIK3CA(E545K)-reverse* signature, a short list of genes predicted to be regulated by either PP or Niclo (Table S2). mRNA analysis from wild type HME cells or isogenic cells carrying the *PIK3CA(E545K)* allele confirmed that these genes were differentially regulated in oncogene-carrying cells compared with wild type control cells (Figure 2A). The treatment with PP or Niclo was sufficient to increase the expression of genes such as *hCCNG2*, *hHBP1*, *hIL8, hHEY1, hTGFA and hCSGALNACT1* that were down regulated by mutated PIK3CA (Figure 2A). In contrast, up-regulated genes such as *hCCNE2*, *hDSCC1*, *hENDOD1* and *hPPP2R1B* were strongly inhibited following PP or Niclo treatments (Figure 2B). PP or Niclo treatments did not change the expression levels of other oncogenic PI3K-target genes, such as *hAXIN1* and *hAXIN2* belonging to the WNT pathway (Figure 2C), thus suggesting a degree of specificity of these drugs towards PI3K target genes. These results demonstrated that: i) PP and Niclo regulate a subset of genes that are under the control of PI3K-dependent pathways; ii) both drugs are able to reverse oncogene-driven gene expression.

### PP and Niclo do inhibit AKT/P70S6K phosphorylation

**Figure 3:**
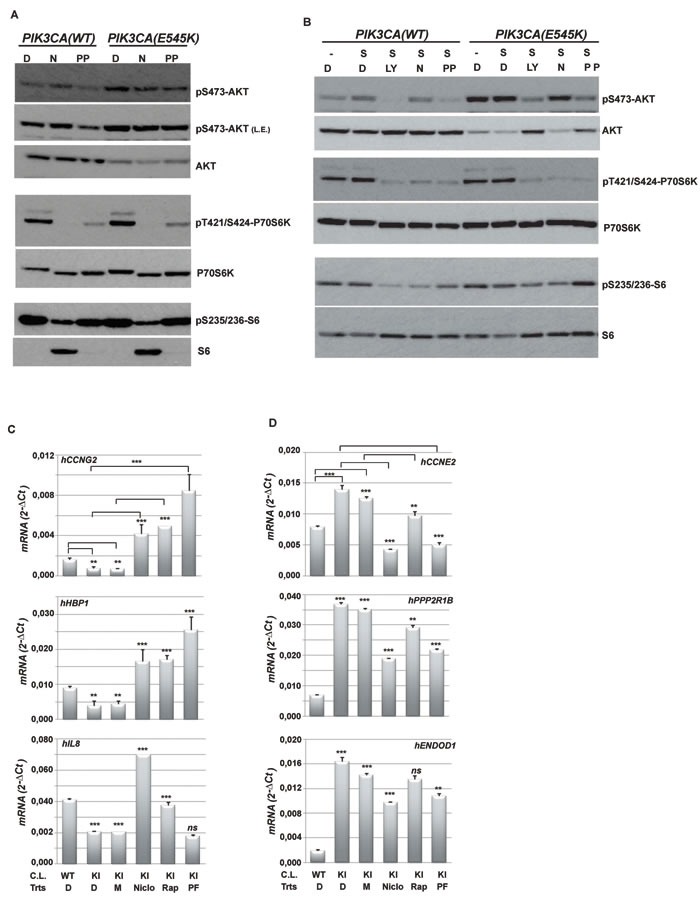
Niclo and PP inhibit the phosphorylation of PI3K-dependent molecular targets **A.** Immunoblot analysis of wild type human mammary epithelia HME cells (PIK3CA-WT) or isogenic cells carrying the oncogenic *PIK3CA(E545K)* mutation (PIK3CA-E545K): cells were treated for two hours with DMSO (D) or Niclo (N) or PP. Cell extracts were analyzed with the indicated antibodies. **B.** Immunoblot analysis of wild type human mammary epithelia HME cells (PIK3CA-WT) or isogenic cells carrying oncogenic *PIK3CA(E545K)* mutations (PIK3CA­(E545K): cells were serum starved overnight and stimulated for two hours with 20% serum (S) prior to harvesting. Equal amounts of protein extracts were analyzed with the indicated antibodies. Before adding serum, cells were pre-treated for 30 minutes with DMSO (D), Niclo (N), PP or LY294002 (LY) inhibitors. Data are representative of at least three independent experiments **C.** and **D.** Total RNA was harvested from wild type *PIK3CA* HME cells (WT) or the isogenic clone carrying the *PIK3CA(E545K)* mutation (KI) after treatments with DMSO (D), methanol (M), Niclo, Rapamycin (Rap) or PF-05212384 (PF) as described in Materials and Methods. Methanol was the vehicle for Rapamycin. The expression of genes down-regulated by oncogenic PI3K (e.g., *hCCNG2*, *hHBP1 and hIL8*) (**C)** or updown-regulated by oncogenic PI3K (e.g., *hCCNE2*, *hENDOD1* and *hPPP2R1B)* (D) was analyzed by quantitative real time PCR. Data indicate absolute values ± SD and are the average of at least three independent experiments. Statistical significance was evaluated through *t-*tests, performed under the following conditions: D-treated KI Vs Niclo-treated KI; M­ treated KI, Vs Rap-treated KI; DMSO-treated KI Vs PF-treated KI. **p < 0.05, ***p < 0.01, ns: not significant.

We investigated the molecular mechanism by which PP and Niclo inhibited PI3K-dependent signalling and, thus, target gene expression. Niclo neither directly bound nor inhibited the p110α subunit of PI3K, as tested by an *in vitro* binding assay (Figure S1). PP was reported in the literature to be inactive against PI3K [34] [39]. We therefore implemented an antigen-capture-based assay to test whether PP and Niclo increased or decreased the phosphorylation and the activation of selected proteins belonging to the PI3K-dependent signalling cascade. We observed that treatment with Niclo significantly decreased P70S6K and S6 phosphorylation in cells carrying oncogenic PI3K (Figure S2). Conversely, PP treatment reduced P70S6K phosphorylation without affecting S6 phosphorylation (Figure S2). We confirmed these results by immunoblot analysis, demonstrating that Niclo treatment strongly inhibited P70S6K and S6 phosphorylation in both wild type and PIK3CA mutated cells (Figure 3A). PP treatment also inhibited P70S6K phosphorylayion but, notably it had a minimal effect on S6 phosphorylation after two hours of treatment (Figure 3A). We also observed a small, but significant, inhibition of AKT phosphorylation by PP. We subsequently evaluated the effect of PP and Niclo treatment during serum stimulation, which is known to activate PI3K-dependent pathways. Under these conditions, AKT, P70S6K and S6 phosphorylation were activated by the PI3K activity, as demonstrated by LY treatment (Figure 3B). Similarly, both PP and Niclo treatment strongly inhibited P70S6K phosphorylation (Figure 3B). In addition, we confirmed that PP did also inhibit AKT phosphorylation, conceivably acting as a dual inhibitor. In contrast to Niclo, PP induced a milder decrease of S6 phosphorylation (Figure 3B): this could be explained through the activation of additional kinases, such as RSK [35] or by varying degrees of P70S6K protein activity inhibition by different compounds. Overall, these results confirmed that both drugs inhibited the PI3K-dependent signalling cascade in cells carrying the *PIK3CA* oncogene.

Results in Figure 3A and 3B indicated that P70S6K or P70S6K and AKT are targets of Niclo and PP, respectively. To formally prove that inhibition of their activities by PP and Niclo was mechanistically linked to the regulation of target gene expression, we pharmacologically inhibited P70S6K by using Rapamycin, a well-known inhibitor of mTOR which is an upstream activator of P70S6K, or PF-05212384 that similarly to PP, acts as a dual inhibitor of both PI3K and mTOR. We then analysed the expression of a subset of genes controlled by PP and Niclo. mRNA analysis demonstrated that Rapamycin was sufficient to increase the expression of *hCCNG2*, *hHBP1* and *IL8* genes in HME cells carrying oncogenic PIK3CA (Figure 3C), as well as in down regulating the expression of *hCCNE2* and *hPPP2R1B genes* (Figure 3D). Moreover, PF-05212384 treatment was sufficient to up-regulate the expression of *hCCNG2* and *hHBP1* and to down regulate the expression of *hCCNE2*, *hPPP2R1B* and *ENDOD1* (Figure 3C and 3D). These results indicated that: i) the selected genes were indeed under the control of AKT/P70S6K-dependent signalling; ii) Niclo and PP regulated PI3K-dependent gene expression, through the inhibition of the AKT/P70S6K axis.

### Anthelmintic drugs inhibit oncogenic PI3K-dependent cellular phenotypes

**Figure 4:**
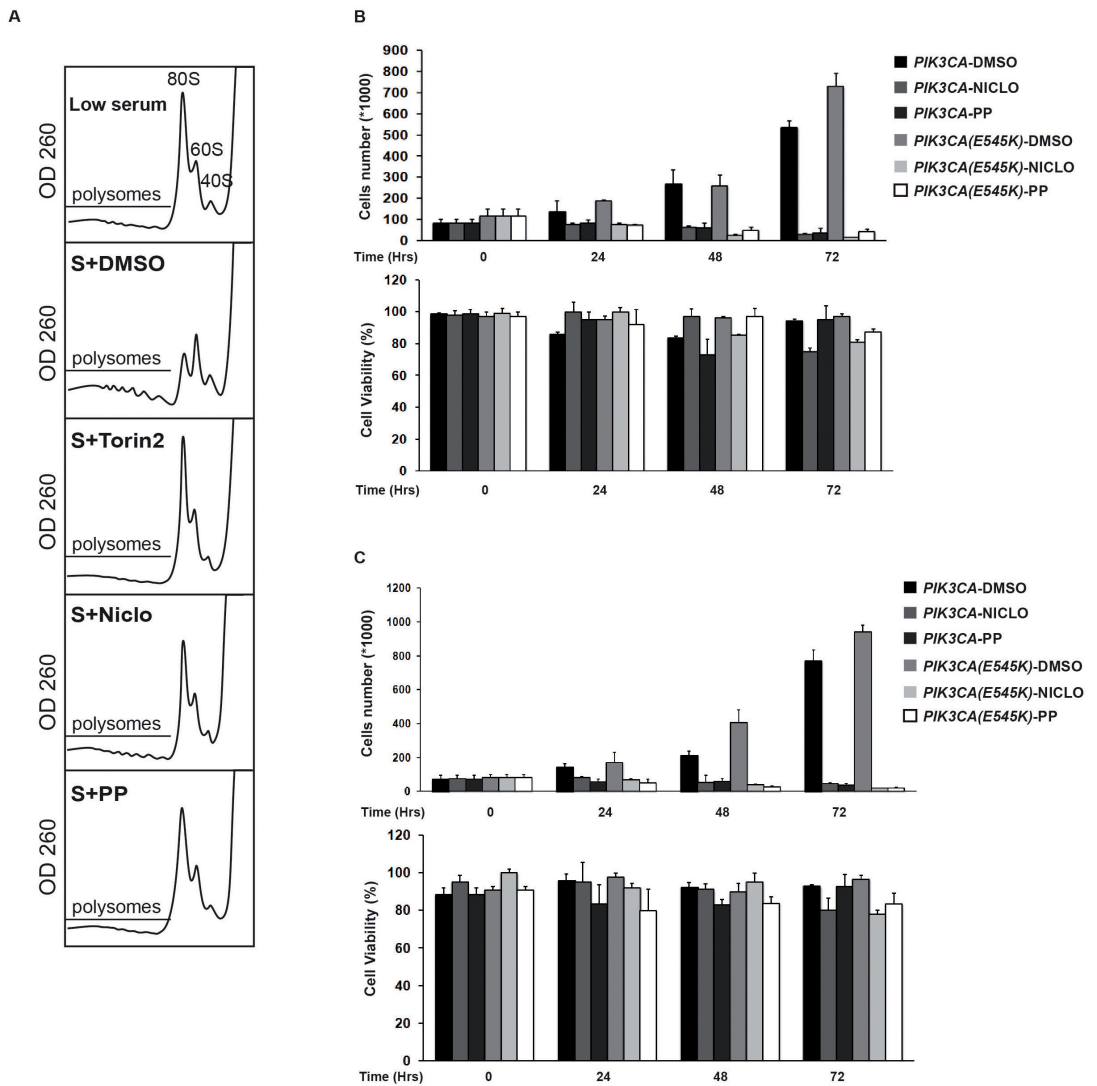
Anthelmintics inhibit oncogenic PI3K-dependent cellular phenotypes **A.** MCF10A-*PIK3CA(E545K)* KI cells were incubated for 24 hours in low serum (0.5% Foetal Bovin Serum)-containing medium, without Insulin and EGF (Low serum). Cells were then stimulated with 5% Foetal Bovin Serum plus Insulin and EGF (S) for 3 hours without or with the indicated drugs. Cytoplasmic extracts were separated on linear sucrose gradients and the absorbance profile at 260 nm was recorded. Region of the gradient corresponding to active polysomes is indicated in each panel. Position of 80S, 60S and 40S peaks is also indicated. **B.**-**C.** Niclo and PP have a strong cytostatic effect. Growth curve of HME (**B)** or MCF10A cells (**C)** carrying wild type PIK3CA or mutated PIK3CA (E545K) treated with Vehicle (DMSO), Niclo or PP. At the indicated time, cell numbers (upper panel) and the percentage of viable cells were calculated compared with non-treated cells. The data are expressed as the mean ± S.D of three independent triplicate experiments.

We next analyzed the effect of anthelmintic drugs on the regulation of biological and cellular processes sustained by AKT/mTOR/P70S6K pathways. A major function of the AKT/mTOR molecular axis is to activate protein synthesis, which it is thought to control through several substrates, including the S6 kinases. ATP-competitive inhibitors of mTOR such as Torin, by blocking canonical mTORC1-dependent events, such as the phosphorylation of S6K1 and 4E-BP1, impair protein synthesis and proliferation [36], [37] [38]. As a step towards defining the ability of PP and Niclo to inhibit AKT/S6K-dependent pathway and their cellular functions in the context of PI3K oncogenic mutations, we examined the effects of PP and Niclo on protein synthesis by scoring for ribosome assembly into polysomes, an early event in protein translation initiation. To focus on the direct translational outputs and avoid secondary effects, we treated cells with inhibitors for only three hours during serum stimulated-protein synthesis. Our results (Figure 4A) showed that, similarly to Torin2, PP or Niclo treatment was sufficient to inhibit the recruitment of ribosomes into polysomes in MCF10A (E545K) KI cells after serum stimulation. These data indicated that PP and Niclo inhibited mTOR and causes a severe defect in translation initiation.

According to the inhibition of protein translation, the inhibition of mTOR/P70S6K in mammary epithelial cells has also a potent cytostatic effect [39]: one might predict that by inhibiting these kinases activities, polysomes assembly and protein translation, Niclo and PP might also elicit cytostatic effects. To test this possibility, we monitored cell proliferation and viability for 72 hours after treatment with Niclo or PP. Our results demonstrate that both drugs had potent cytostatic effects (upper panel), with very minimal cytotoxicity (lower panel) in HME (Figure 4B) and MCF10A cells (Figure 4C). As expected, Niclo and PP treatment have also cytostatic effects in isogenic cells carrying a constitutively active, mutant PIK3CA.

**Figure 5:**
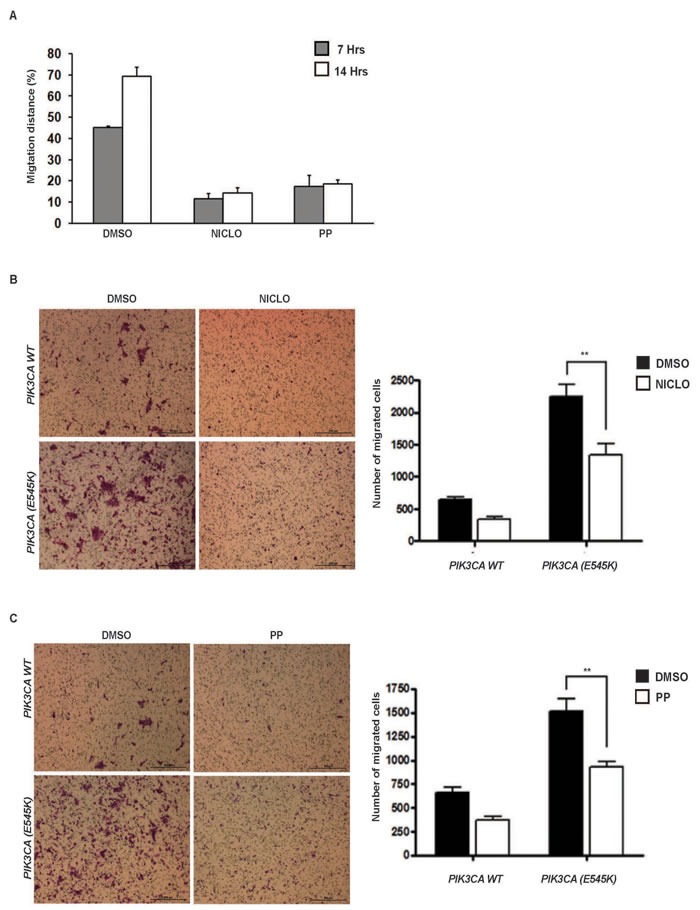
Niclo and PP inhibit oncogenic PI3K-mediated cellular migration **A.** *In vitro* wound healing assay of MCF10A-*PIK3CA(E545K)* mammary cells kept in EGF-free serum. Percentage of migration distance referred to total distance was calculated at 7 hrs and 14 hours after *in vitro* scratch, in presence of DMSO, Niclo or PP. Data indicate average ± SD. **B.** and **C.** A transwell assay measuring cell migration of MCF10A cells with or without *PIK3CA(E545K)* in the presence or absence of Niclo (10μM) (**B)** or PP (3.4 μM) (**C)**. The number of migrated cells is shown as the mean and SEM of six biological replicates. *P*-values are calculated by t test: *p < 0.05, **p < 0.005, *** *p* < 0.0005, unmarked > 0.05.

The oncogenic activation of PI3K-dependent signalling in mammary cells supports cellular motility, invasion and Epithelial-Mesenchymal Transition (EMT) [27, 40-41]; moreover, PI3K/mTOR pathways accelerates wound healing [42]. We observed that EGF-deprived wild type MCF10A, but not MCF10A-*PIK3CA(E545K)* cells showed a 50% reduction in migration in a scratch wound healing assay indicating that, under such conditions, cell motility was activated by oncogenic PI3K signalling (Figure S3). We thus tested the ability of Niclo or PP to inhibit wound healing when selectively activated by oncogenic PI3K signals in EGF-deprived cells. The treatment with Niclo or PP was sufficient to completely inhibit the migration of MCF10A-*PIK3CA(E545K)* cells after 14 hours (Figure 5A). We also tested the ability of anthelmintic drugs to suppressing PI3K-dependent cellular invasion following stimulation by attractant hormones. Our results showed that MCF10A-*PIK3CA(E545K)* cells had almost a 2.5 fold increase in cells invasion compared with wild type isogenic cells (Figure 5B-5C). Treating cells with Niclo or PP significantly reduced, about 50%, the invasion of cells carrying the *PIK3CA(E545K)* mutation (Figure 5B-5C).

Collectively, these data demonstrate that PP and Niclo inhibited oncogenic PI3K-dependent cellular phenotypes. This confirms a successful repositioning of both drugs as negative regulators of constitutively active PI3K-dependent pathways.

### PP controls mammary branching morphogenesis and PI3K-dependent signaling of mouse mammary gland tissue

**Figure 6:**
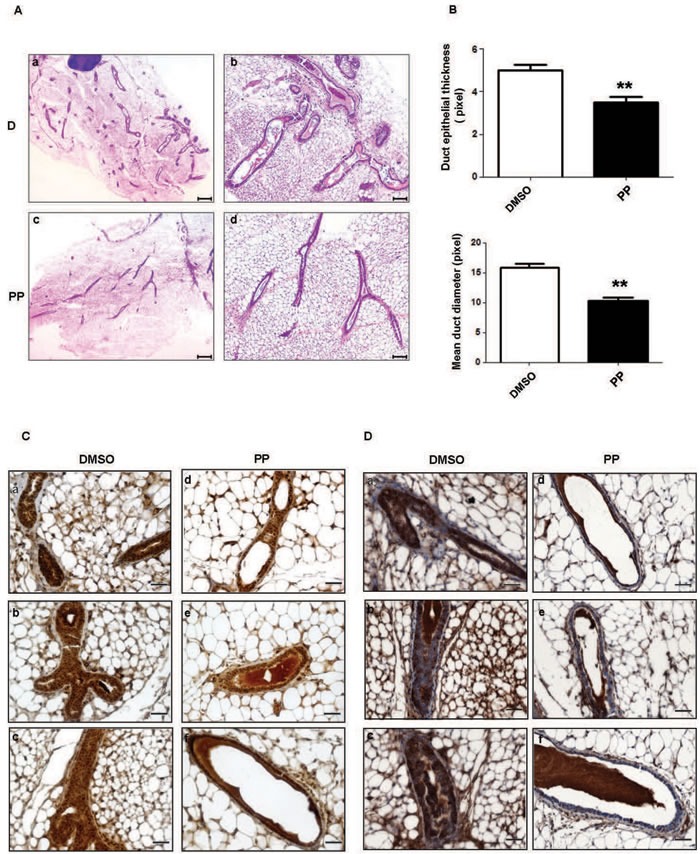
PP controls mammary branching morphogenesis and PI3K-dependent signaling in mouse mammary gland tissue **A.** Representative H&E stained sections of mammary glands from female mice treated with DMSO (D) (panels a-b) or PP (panels c-d). a and c: images at 2,5x magnification; b and d: images at 10X magnification. Images are representative of different fields of gland sections derived from three different mice *(n = 3).* **B.** Duct epithelial thickness (upper panel) and ducts diameter (lower panel) were analyzed in H&E stained sections of mammary gland from female mice treated with DMSO or PP. Means ± SEM are shown in histograms. P-values were calculated by t test: ** *p* < 0.05. **C.**-**D.** IHC analysis of mammary gland sections of female mice treated with DMSO (panels a-c) or PP (panels d-f) and stained with anti phospho-AKT (**C**) or anti phospho-S6 antibodies (**D**) Images are representative of different fields of gland sections derived from three different mice *(n = 3).* Images were captures at 40x magnification; images at 4x and 20x magnification were also analyzed (Figure 5S). Scale bars: 400 μM (a and c H&E images), 100 μM (b and d H&E images), 30μm (IHC images).

We next tested the effect of anthelmintic drugs in controlling proliferation and targeting the PI3K/P706SK-dependent pathways *in vivo,* and we focused on PP. Since in *vitro* studies were performed with mammary epithelial cell lines, we assayed the effect of PP treatment in mammary gland tissue of mice. Mammary ductal morphogenesis depends on PI3K-dependent signalling and PI3K activation has been directly correlated to the control of ductal branching and alveolar cell proliferation during mammary gland development [43]. To investigate if PP, by inhibiting PI3K-dependent signaling *in vivo,* might suppress duct morphogenesis, we analyzed mammary gland tissue after PP treatment. Haematoxylin stained mammary gland tissue sections showed thinning ducts and a significant reduction of duct diameter in PP-treated compared to control-treated mammary glands (Figure 6A-6B), thus confirming a cytostatic effect of PP *in vivo* and a reduction in mammary branching morphogenesis. Immunohistochemical analysis (IHC) of mammary gland tissue sections revealed that PP effectively reduced the level of S6 and AKT protein phosphorylation compared to vehicle treated glands (Figure 6C-6D, Figure S5). Notably, a substantial reduction in protein phosphorylation was observed in both duct epithelial cells and mammary gland-associated adipocytes.

### PP inhibits growth of breast cancer cells harbouring *PIK3CA* mutations

**Figure 7:**
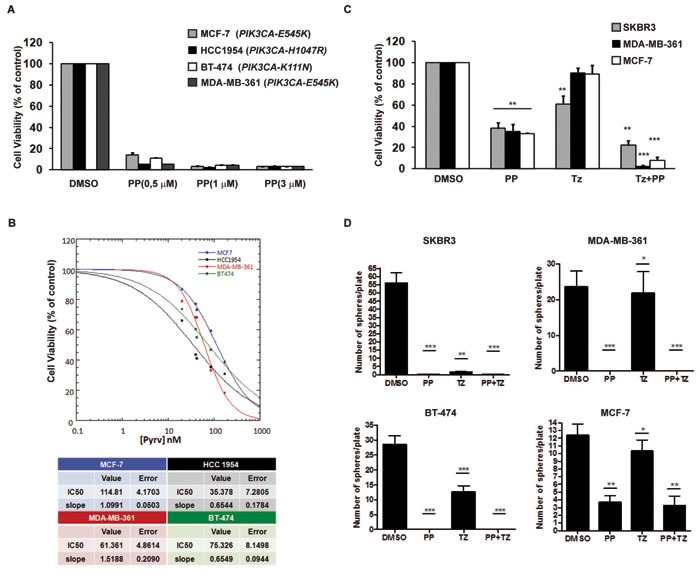
PP inhibited the growth of breast cancer cells carrying *PIK3CA* mutations **A.** Breast cancer cell lines carrying the indicated PIK3CA mutation were seeded in 96-well plates. DMSO or Pyrvinium Pamoate (PP) was added at indicated concentrations and cell viability was evaluated after 72 hours by the CellTiter-Glo assay. The results are the mean ± SD of 3 independent experiments showing the percentage of cells viability over that of DMSO-treated cells. **B.** Upper panel: Representative graphs from Kaleidagraph software showing dose-response curves to Pyrvinium Pamoate (20 to 200 nM) in different cancer cell lines. Dose is presented on the x axis, while cell viability (% vs control) is presented on the y axis. Lower panel: Tables showing IC50 and slope values. **C.** and **D.** HER2 positive (HER2+) cancer cell lines (MDA-MB-361 and BT-474) carrying PIK3CA mutations were treated with PP at the IC50 concentration or with Trastuzumab (Tz) alone (10μg/ml) or in combination (Tz+PP). The Transtuzumab-responsive SKBR3 cell line (HER2+/PIK3CA Wild-Type) and Transtuzumab-resistant MCF7 (HER2-/PIK3CA(E545K)) were used as positive and negative controls, respectively. **C**. Cell viability after 72 hours was evaluated as in A **C.**. **D.** Soft agar colony forming assays were carried out with the indicated cancer cell lines. 1000 cells were seeded per well (2000 for BT-474) and treated with PP (at IC50 concentration) or Tz alone (10μg/ml) or in combination with PP. Tz was added once a week for 2 consecutive weeks. Colonies were stained with crystal violet at ∼4 weeks after initial seeding. Statistical analyses were performed using GraphPad Prism version 4 (www.graphpad.com). Statistical significance was determined by one-way ANOVA *P* value < 0.05*, *P* value < 0.01**, *P* < 0.001***.

Oncogenic mutations in the PI3K pathway are frequent in breast cancers and support cancer growth as well as resistance to HER2 targeting agents: PI3K or dual PI3K/mTOR inhibitors are able to kill breast cancer cells [18] and have demonstrated to have clinical benefits for the treatment of breast cancer carrying PI3K mutations [44]. We investigated if PP, by virtue of its dual AKT and P70S6K inhibitor activity, inhibited the growth of cancer cells harbouring PI3K mutations. Our results demonstrated that PP treatment was highly cytotoxic against breast cancer cells with an average inhibitory concentration 50 (Ic50) of 50nM (Figures 7A and 7B). Since PI3K mutations in HER2-positive tumors is an important determinant of resistance to anti HER2 therapy Trastuzumab [45], we next tested the ability of PP to kill such resistant cancer cells compared with Trastuzumab alone or in combination. Compared with Trastuzumab, PP treatment inhibited the growth and soft agar colony formation of Trastuzumab-resistant cancer cells (Figures 7C and 7D). Together, these data indicated that anthelminthic PP might represent a valuable pharmacological inhibitor of breast cancer cells carrying PI3K mutations.

## DISCUSSION

We propose a paradigm for exploiting large databases of transcriptional genes signatures for computational repositioning of drug inhibitors against a specific oncogenic pathway, by identifying drugs able to revert the associated oncogenic gene signature. The *in-silico* identification of well-known inhibitors of the PI3K-dependent pathway such as Sirolimus (rapamycin), LY-294002, Wortmannin and Quinostatin demonstrates the efficacy of the methodology to identify PI3K-dependent pathway inhibitors and to potentially reposition novel compounds.

In this study, the reference signature for oncogenic, active PI3K-dependent pathways had been derived by using isogenic KI human mammary epithelial cells expressing the *PIK3CA(E545K)* or *PIK3CA(H1057R)* cancer alleles, that are suitable for generating bona-fide oncogene specific signatures. Several studies have demonstrated the potential of using gene expression profiles of cancer cells for the analysis of oncogenic pathways, and such profiles can reflect deregulation of specific pathways in cancers [3, 46]. Thus, our approach could potentially be exploited for the repositioning of drugs targeting oncogenic deregulated pathways identified by tumor-derived signatures. It is worth observing that oncogenes transcriptional outputs might differ among different tissues and this could explain the different role of oncogenic pathway among different tumors. Moreover, the cooperation among oncogenes and tumor suppressors genes, frequently occurring with tissue specificity, should be also considered. Thus, the transcriptional output of oncogenic signalling might have a certain degree of tissue specificity.

We demonstrated that nodes generated by oncogene-induced signatures or inhibitor-induced signatures are both efficient in searching for computational similarities in drug networks; however, combining both approaches would be extremely powerful, by increasing stringency for drugs discovery and by limiting the likelihood of false positive. If available, the inhibitor-induced signatures can assist the oncogene signatures-based approach in increasing the efficiency of computational repurposing methods. In the case of the PI3K pathway, the oncogene-induced signatures approach is sufficient to the computational repurposing of novel pathway inhibitors. This demonstrates the strength of the approach presented, because pathway-specific gene signatures could be generated with high efficacy by genetic manipulation (e.g., by means of somatic KI or Knock-out targeting approaches). In contrast, pharmacological inhibitors might not be available at times, as in case of “undruggable” targets. Thus, the approach presented in the manuscript could be exploited and applied to every genetically targetable oncogenic pathway.

The herein proposed drug repositioning identifies novel potential therapeutics, PP and Niclo to be further investigated for the treatment of human diseases showing a constitutive activation of PI3K/mTOR/P70S6K signalling axis. Notably, inhibitors identified by means of genetic signatures might offer opportunities to target aberrant transcriptional signatures regardless of the specific genetic lesion causing PI3K constitutive activation and thus be effective for a larger number of PI3K-dependent pathological phenotypes. Moreover, the inspection of network communities close to PI3K inhibitors might help to predict PI3K inhibitor off-target effects and, possibly, side effects.

What molecular mechanisms link Niclo and PP to PI3K-dependent pathway inhibition? Niclo and PP do not directly bind and inhibit the catalytic subunit of PI3K implying that additional or indirect mechanisms should be considered. Niclo has been previously recognized as a protonophore molecule that decreases intracellular pH by extruding protons from lysosomes, thus lowering cytoplasmic pH. This, in turn, inhibits mTORC1 signalling [47-48] and, consequently, may inhibit P70S6K phosphorylation. In agreement with our data, previous drug screens identified Niclo as a negative regulator of P70S6K, without AKT inhibition [47, 49]. Conversely, analysis of structure and reactivity similarity (Figure S4) indicated that PP profile is not as protonophore. PP has been proposed to target mitochondrial complex I [30], thus reducing oxidative phosphorylation rate and ATP production. PP treatment might produce a starvation-like condition resulting in the activation of starvation-related sensors such as AMPK, which negatively regulate both mTOR and P70S6K phosphorylation [36]. In this scenario, both PP and Niclo would act as indirect inhibitor of P70S6K activity, although further molecular studies are needed to delineate the mechanism.

In conclusion, irrespective of the PI3K pathway, we believe that our approach represents a paradigm that can complement drug discovery approaches for undruggable molecular targets or aid in cases in which traditional high-throughput drug screens of inhibitors against selective signalling pathways are likely to fail.

## MATERIALS AND METHODS

### Cell lines

Mammary epithelial hTERT-HME1 (HME), MCF10A and isogenic derivatives carrying specific cancer alleles have been described previously [26] and were cultured as described [26]. The MCF7, HCC1954, BT-474, MDA-MB-361 and SKBR3 cell lines were obtained from ATCC and cultured according to the provider's instructions.

### RNA target preparation/affymetrix microarray studies

Gene expression analysis was carried out using RNA extracted from sub-confluent cell cultures using an Invitrogen kit. RNA samples of sufficient quality were profiled on Affymetrix Human Gene 1.0 chips. Preparation of complementary RNA, array hybridizations, scanning and subsequent array image data analysis were done using the manufacturer's specified protocol.

#### Evaluation and normalization of affymetrix genechip data and generation of reverse signature

The data were analyzed with the Bioconductor package “Affy” using the RMA method for normalizing and summarizing probe level intensity measurements. The profile “1” (HME­PIK3CA(E545K) - Reverse signature) had been processed before to make it compatible with the standard of Mantra. We have developed a method to convert “Affymetrix Human Gene 1.0” probes into Affymetrix HG-U133A probes (chip reference for the network of Mantra). The conversion was carried out in two steps: 1) from “Affymetrix Human Gene 1.0” probe_set_id towards “Affymetrix Human Gene 1.0” gene: each probe set id was associated with the corresponding gene, based on Affymetrix annotation. If more than one probe was found to be associated with the same gene, we assigned to the gene the rank of the probe that comes first in the ranking. 2) from “Affymetrix Human Gene 1.0” gene towards “Affymetrix HG-U-133A” probe_set_id: we associated to each gene the corresponding HG-U-133A probe set, using the Affymetrix annotation file. If a gene was associated with more than one probe set, we assigned the same ranking to all of the probes involved. For Affymetrix probes that did not generate any matches we positioned them at the center of the ranking. We then integrated them in the drug network as previously described [23]. The other two profiles were obtained from Array Express ID “E-GEOD-17785”. In this case the chip HG-U133_Plus_2 was already compatible with the reference chip of Mantra.

### Mantra 2.0 analysis and community enrichment

Gene expression profiles were compared to those previously generated for 1,309 compounds (cMAP) [22] by computing the transcriptional similarity between our profiles and each of the 1,309 compounds as previously described [7]. Specifically, Affymetrix microarrays. CEL files were uploaded in the MANTRA on-line tool (http://mantra.tigem.it) and automatically transformed into a new node in the drug network [24]. MANTRA computed the transcriptional similarity between each treatment and each of the 1,309 compounds in cMAP. The transcriptional similarity is quantified as a distance, which is a number greater or equal to zero, with zero indicating identical profiles [7]. We then selected all drugs that scored below a transcriptional distance threshold of 0.86 from all our profiles. A set of 27 drugs were thus selected to be significantly similar to our signatures. Community enrichment analyses were performed as previously described [7]. According to the neighbors of each profile a hypergeometric test was applied and a *pValue* threshold of 0.05 was used to identify the communities more enriched for each profile.

### Cell viability and soft agar colony assay were performed as previously described [26]

mRNA expression analysis, protein extraction, western blot analysis, polysomes assembly analysis, animal studies, tissue collection, histology and immunohistochemistry methods and structure overlaps are described in the Supplementary Materials.

### Statistical analysis

Where appropriate, specific statistical analysis and approaches have been described in the figure legends.

## SUPPLEMENTARY MATERIAL FIGURES




