# Supplementary material for: Computational drugs repositioning identifies inhibitors of oncogenic PI3K/AKT/P70S6K-dependent pathways among FDA-approved compounds
Source: Oncotarget. 2016 Aug 16;7(37):58743–58. doi: 10.18632/oncotarget.11318 (PMC5312272; doi:10.18632/oncotarget.11318)
Supplement: Supplementary file 1 [file oncotarget-07-58743-s001.pdf]

Computational drugs repositioning identifies inhibitors of oncogenic PI3K/AKT/P70S6K-dependent pathways among FDA-approved compounds

Supplementary Material

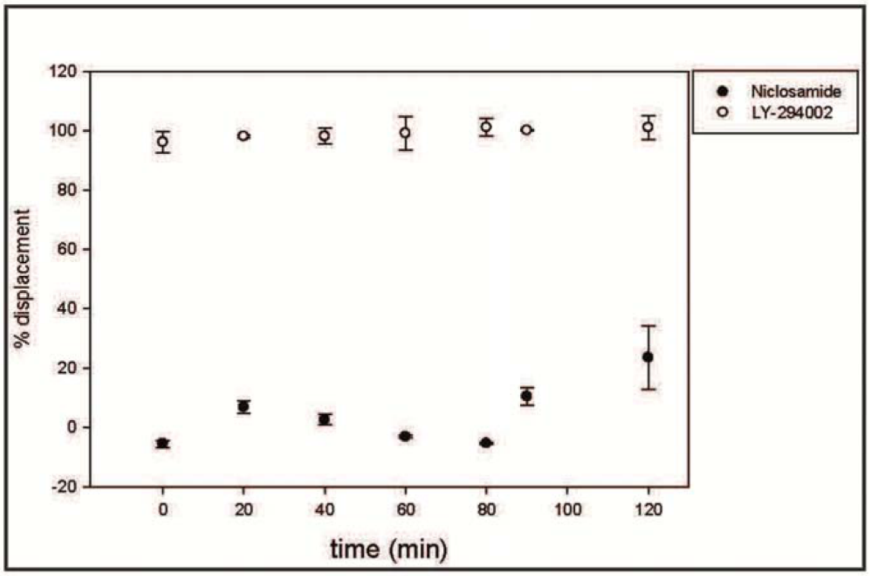

FIGURE S1

Figure S1. Niclo does not inhibit probe binding to the PI3K active site. A Fluorescence Resonance Energy Transfer (FRET)-based assay was used to measure in vitro inhibitor competition for probe binding to the PI3K active site (LanthaScreen Eu Kinase Binding Assay, Invitrogen). Compounds were tested at 10 µM concentration. The values represent the means of three independents replicates. The figure shows % displacement of the tracer over time. Full displacement was observed for LY-294002, used as positive control, from the first time point, which is maintained over time. No displacement was observed for Niclosamide up to 100 min.

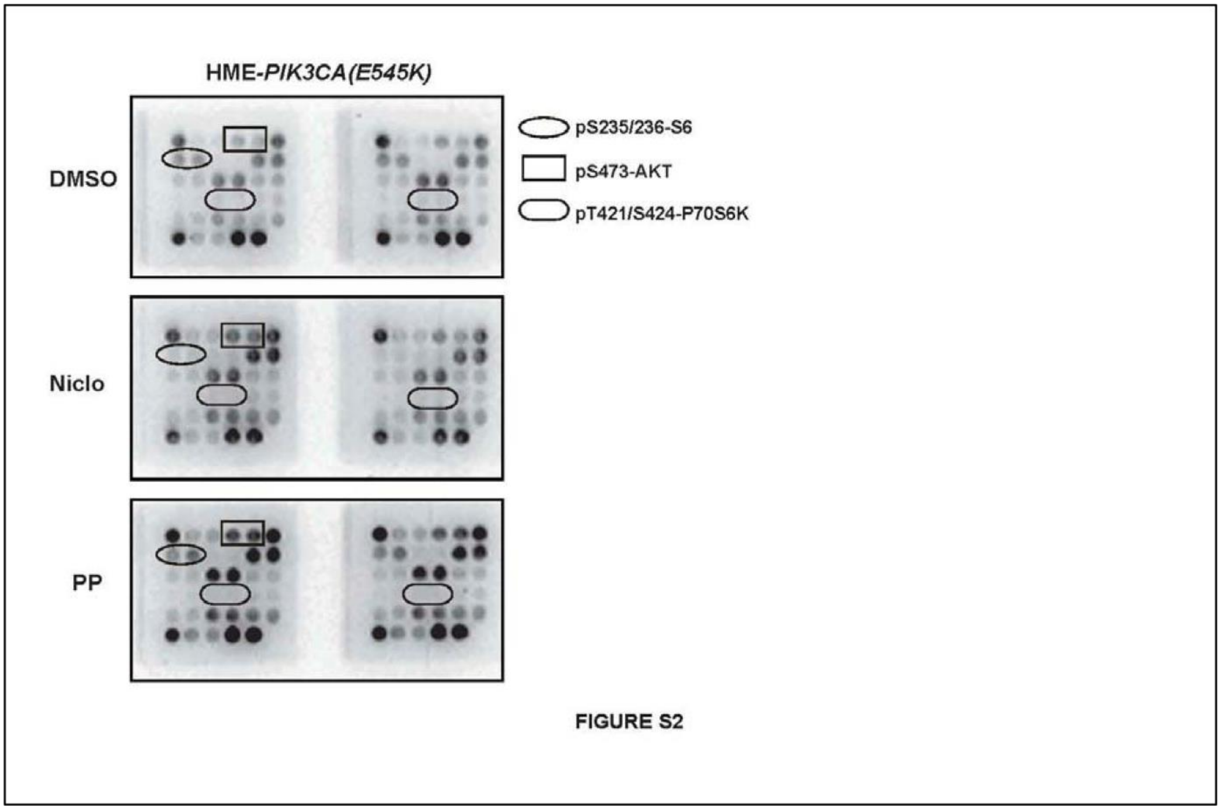

Figure S2: Antigen capture-based antibody array identified potential molecular targets of PP and Niclo. HME cells carrying the PI3K (E545K) mutation and treated with vehicle (DMSO), Niclo (10µM) or PP (3.4 µM) for 2 hours. Protein lysates were analyzed with the PathScan antibody array for the phosphorylation status of 18 central phosphorylation residues of signaling targets and nodes belonging to the PI3K/AKT signalling. The AKT, S6 and P70S6K phosphorylated signals are circled and presented as duplicate. Each experiment was performed as independent duplicated and shown in left and right arrays. The whole array targets map is available through the manufacturer's homepage: http://www.cellsignal.com (ref.# 9474).

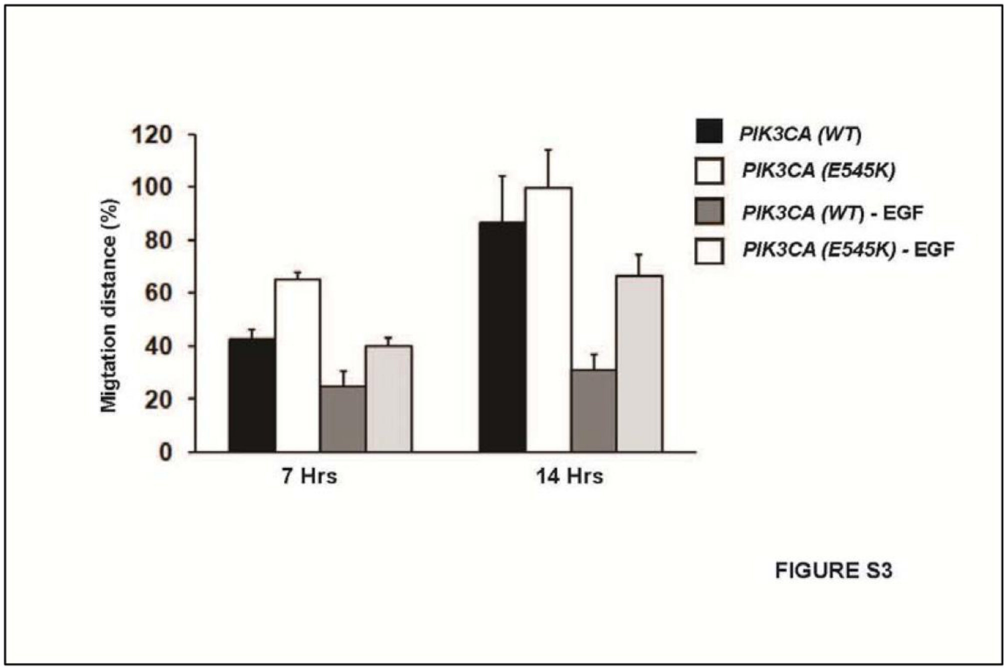

Figure S3. Oncogenic PI3K drive migration of mammary cells in wound healing-based assay. MCF10A cells were kept in EGF-free medium: under such treatment, only cell carrying oncogenic *PIK3CA(E545K)* mutation promote wound healing

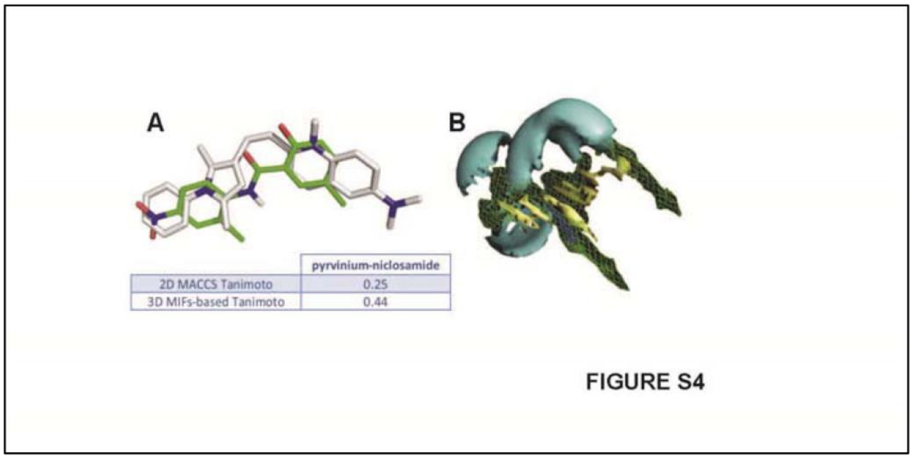

Figure S4. 2D and 3D structural alignments of PP and Niclo. The best 2D **(A)** and 3D **(B)** structural alignment pose of PP and Niclo according to the highest FLAP GLOB-S similarity score (0.44). In panel B, the MIFs overlapping is rather low, especially for the polar interaction given by the probe N1 (H-bond acceptor interaction; cyano surface) of Niclo (DN1=0.0). Regarding hydrophobic interaction detected by the DRY probe, a small overlapping of the yellow and black/green mesh surfaces (DDRY=0.23) was observed, while the shape overlapping of the two drugs seems to be higher (Dshape=0.54). Nevertheless, the global 3D structural similarity (GLOB-P score) is 0.44, indicating that the two drugs are not structurally similar. This results is in agreement with the 2D topological similarity analysis with the MACCS key fingerprints as well (D2D=0.25) **(A).**

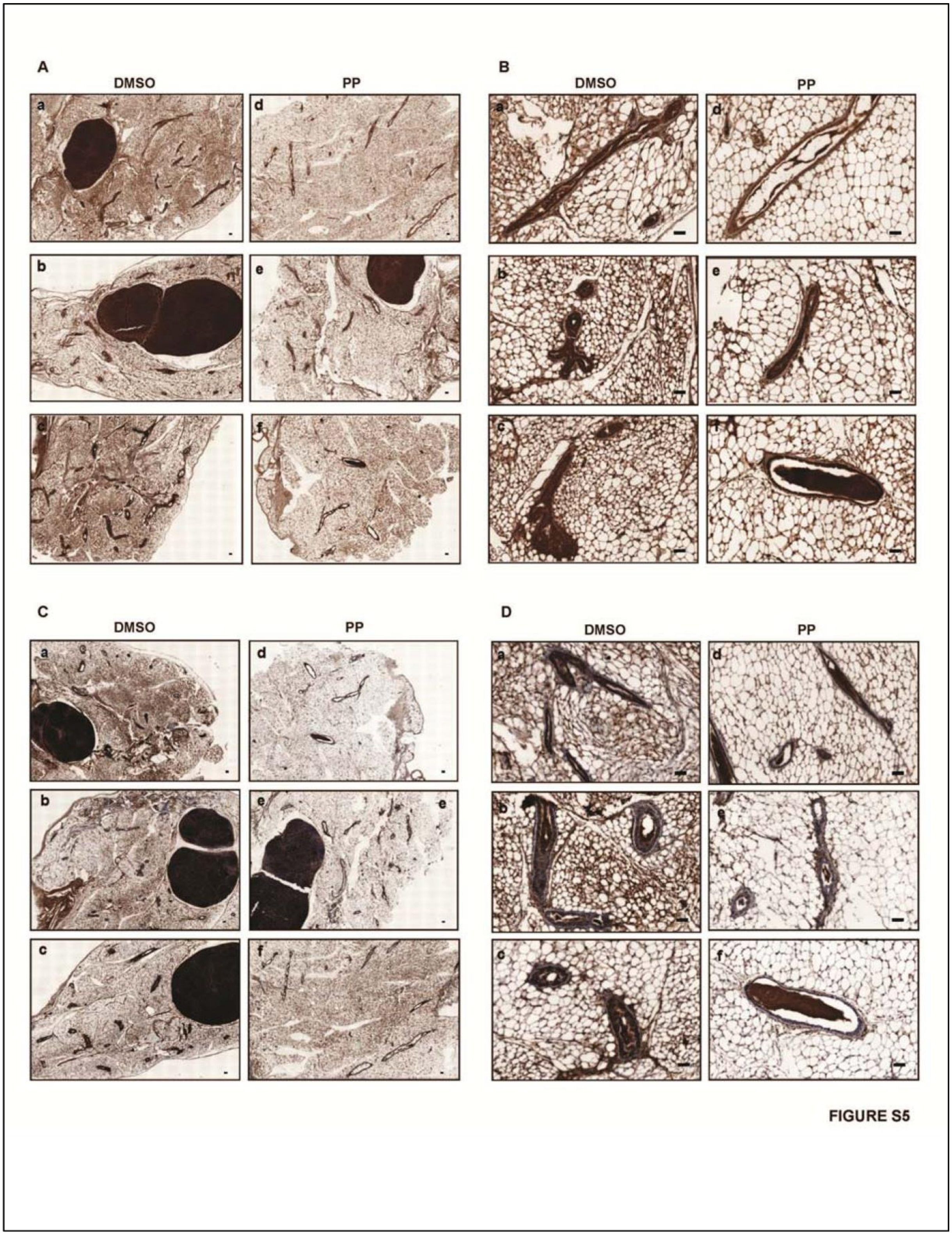

Figure S5. PP controls PI3K-dependent signaling in mouse mammary gland tissue. (A-B) IHC analysis of sections derived from mammary gland of female mice treated with DMSO (panels a-c) or PP (panels d-f) and

stained with anti phospho-AKT antibody. Images were captures at 4x **(A)** and 20x magnification **(B). (C-D)** IHC analysis of sections derived from mammary gland of female mice treated with DMSO (panels a-c) or PP (panels d-f) and stained with anti phospho-S6 antibody. Images were captures at 4x **(C)** and 20x magnification **(D).** Each image is representative of different fields of glands sections from three different mice *(n=3).* Scale bars: 30μm.

Supplementary Material and Methods

Antibodies and Reagents

The following antibodies were purchased from Cell Signaling Technology and used according manufacturer's instructions: anti phospho-AKT(Ser473) (ref.: #4060), anti AKT((ref.: #9272), anti P70S6K(ref.: #9202), anti phospho-P70S6K(Thr421/Ser424) (ref.. #9204), anti phospho-S6(Ser235/236) (ref.: #2211) and anti S6 (ref. : #2317). The anti-β-actin antibody (ref.: #A2228) was purchased from Sigma-Aldrich. PF-05212384, LY-294002, Pyrvinium Pamoate and Niclosamide were purchased from Sigma-Aldrich. Rapamycin was purchased from Cell Signaling Technology.

Cell Proliferation and viability assay

Cells were plated at a density of 47000 $cells/cm^2$ in triplicate in 6-multiwell plates in complete culture medium with or without the relative drug: Niclosamide [10□M], Pyrvinium [3,4 μM]. Once adherence was reached, 3 wells/cell lines were washed with PBS and detached incubating for 5 minutes at 37 degrees with ATV. The enzymatic reaction was brought to an end with complete culture medium and an aliquot from each well was diluted 1:2 in trypan blue and loaded on a hemocytometer to be counted. This count has been considered the starting point (T=0). The same thing has been repeated for each time point.

Polysome analysis

About 1,1x10 [6] cells were plated. After 24 hours cells were washed twice with serum and hormones-free medium and treated with low serum containing medium (0,5% Foetal Bovin serum, 0,5 μg/ml hydrocortisone). After 24 hours cells were left untreated pr pre-incubated for two hours with DMSO (vehicle) or Torin2 (0.1 nM) or Niclo (10 μM) or PP (3.4 μM), before being stimulated for three hours with 5% serum plus Insulin (10μg/ml) and EGF (20ng/ml), in presence or not of inhibitors. Cell lysis and sucrose gradient sedimentation were performed essentially as previously described (Caldarola et al., 2004). Briefly, cells were washed once with phosphate-buffered saline buffer (150 mM NaCl, 2.7 mM KCl, 8 mM NaH2PO4 and 1.4 mM K2PO4) and then lysed directly on the plate with 300 μl of lysis buffer (20 mM Tris-HCl, pH 7.5; 10 mM NaCl; 10 mM MgCl2; 1% Triton X-100; 1% Sodium deoxycholate; 5 mM DTT; 0.13U/μl RNasin; 50 mM NaF; 1 mM Na3VO4; 1 mM Benzamidine; 10 mM β-glycerophosphate; 1 mM PMSF; protease and phosphatase inhibithors cocktail). Cell extracts were collected in eppendof tubes and centrifuged for 10 min at 4°C in a microcentrifuge at 16 000 g. Pellet was discarded and heparin (30 μg) was added to the supernatant (cytoplasmic extract) to further avoid RNA degradation. Cytoplasmic extracts were layered onto 11 ml of 15-50% of linear sucrose gradient containing 30 mM Tris-HCl, pH 7.5, 100 mM NaCl, 10 mM MgCl2, and centrifuged at 234 000 g for 2h and 15 min at 4°C in a Beckman SW41 rotor. The 260 nm absorbance profiles of the samples were measured with an LKB Bromma 2238 Uvicord SII.

Wound Healing assay

Cell migration was assessed by a wound healing assay. Cells were cultured in 12-well plates until confluent rate reached 90-95% and then starved ON in a 0,5% FBS supplemented medium without EGF. Eighteen hours later the cell layer was wounded using a 200 μL sterile tip and a new starvation medium was added to each well, with or without the relative drug: Niclosamide [10μM], Pyrvinium [3,4 μM]. The spread of wound closure was observed and photographed through a time lapse microscope (Nikon Eclipse, Nikon Instrument BV, Amsterdam, NL) set for shooting an image every 20 minutes. Tests were carried out two times.

Transwell-based migration assay

Migration assay was performed plating cells in transwell permeable supports (Corning Incorporated, Corning, NY) according to the manufacturer's instructions. Briefly, after cultured in complete culture medium, cells were cultured 24 hours in starvation medium (complete culture medium diluted 1:10 in DMEM/F12 unsupplemented medium containing L-glutamine and antibiotics). After 24 h of starvation, 10000 cells were resuspended in 100 μL of starvation medium with or without the relative drug: Niclosamide [10□M], Pyrvinium [3,4 μM] and plated onto inner well. In the lower part of the chamber 700 μL of complete culture medium was added. After 24 h of incubation, the cells that invaded through the membrane was fixed and stained with crystal violet PBS solution (0.05% w/v crystal violet, 1% formaldehyde, 1% methanol). The number of cells that invaded were counted under light microscope with a 4X magnification

In vitro p110*α*/p85 catalytic assay

A Fluorescence Resonance Energy Transfer (FRET)-based assay was used to measure in vitro binding of the compounds to the p110a subunit of PI3K (LanthaScreen Eu Kinase Binding Assay, Invitrogen). Binding of the tracer (Alexa Fluor® 647-labeled probe) to p110 is detected by addition of an Eu-labeled anti-tag antibody. Simultaneus binding results in a high degree of FRET. Competitive binding to p110 by inhibitors results in a time-dependent displacement of the tracer. Nicosamide and LY-294002 (used as positive control) were analysed at 10μM concentration. Experiments were done in three independent replicates. Pyrvinium Pamoate could not be analysed due to interference with the assay.

Identification and selection of most regulated genes

PIK3CA(E545K)-reverse-, PP- and Niclo-dependent signatures were analyzed by Mantra to identify the 250 most upregulated and downregulated genes in the HG-U133A Affymetrix array. Among these selected genes, commons between PIK3CA(E545K)-reverse- and Niclo-dependent signatures or PIK3CA(E545K)-reverse- and

PP-dependent signatures were selected for the experimental validation.

mRNA expression analysis.

Total RNA extraction was carried out using Rneasy kit (Quiagen) according to the manufacturer's instructions. Total RNA was then reverse-transcribed into cDNA by QuantiTect Reverse Transcription Kit (Quiagen) with random hexamers oligo. The cDNA was diluited 1:3 and subjected to quantitative real time PCR analysis by using Light Cycler (Applied Biosystem) with SYBR Green PCR Master MIX Kit (Applied Biosystem). The primer sequences for the PCR analysis are available on request.

Protein extracts and Western blot analysis.

Cells were washed in phosphate-buffered saline 1X (PBS1X) containing 1X protease inhibitor cocktail and 1mM PMSF and were lysated in modified radioimmunoprecipitation assay buffer (RIPA-1: 50 mM Tris-HCl [pH 7.8], 150 mM NaCl, 5 mM EDTA, 15 mM MgCl2, 1% Nonidet P-40, 0.5% sodium deoxycholate, 1 mM dithiothreitol, 1X protease inhibitors, 1mM PMSF, 50mM NaF, 10 mM β-glycero-phosphate and 1 mM Na3VO4, and then immediately frozen in liquid nitrogen. Cleared protein extracts were quantified by using the Bradford method (Bio-Rad). For western blotting, protein samples were separated on 8%–12% SDS-PAGE and transferred to nitrocellulose membrane (Amersham). Membranes were blocked in TBS containing 5% bovine serum albumin, incubated with primary antibodies according to the antibody manufacturer's instructions, followed by incubation with horseradish peroxidase-conjugated goat anti-rabbit or anti-mouse IgG (Amersham) and enhanced chemiluminescence detection (Pierce).

Animal studies and tissue collection

Six-week old female C57BL/6 mice (Charles River Laboratories) feed *ad libitum* were administered with PP (0,1mg/kg/dose) or vehicle only (DMSO) diluted in physiological solution. Solutions were made fresh at every days of treatment and administered by injection into inguinal mammary gland. Doses were achieved based on mouse weight; treatments were done on day1 and day4 /week. After 3 weeks, the animals were bled and euthanized. Mammary glands were collected from each mouse and preserved in 10% buffered formalin overnight at 4°C, transferred to 70% ethanol and stored at room temperature until sectioning. The Institutional Animal Care and Use Committee (IACUC) approved all mouse experiments.

Histology and Immunohistochemistry

Formalin-fixed paraffin-embedded samples from mouse mammary glands were sectioned for Hematoxylin and Eosin staining and immunohistochemistry. After antigen retrieval at 96°C (10 mM/L citrate buffer, pH 6) for 20 minutes, 2 micron sections were incubated with monoclonal antibodies anti-phosphoAkt-1 (Ser473) 1:50 O/N (Cell Signaling) , anti-phospho S6 (Ser235/236) 1:150 (Cell Signaling) for 30 minutes at room temperature. Immunoreactions were revealed by Bond Polymer Refine Detection Kit according to manufacturer's procedure (Leica, Milan, Italy). Diaminobenzidine was used as chromogenic substrate and slides were counterstained with haematoxylin, dehydrated and coverslipped. Images were obtained at 4x, 10x and 40x magnification by using a light microscope (Nikon, Eclipse 55i) equipped with a software able to capture images (Eureka Interface System). Digital pictures in Figure 6 A were captured with a Leica DC500 camera and a 2.5x objective. Glands larger than a single imaging area were captured by photographing contiguous microscopic fields in a raster pattern. Each captured image was merged using the layer technique in Adobe Photoshop to form a single composite image for analysis.

Duct diameter and duct epithelial thickness were evaluated with Adobe Photoshop by measuring the larger trasversal diameter and the thickness of the epithelial layers in the same point with the ruler tool.

Measurements were recorded on an Excel spreadsheet and mean and SEM were calculated for both groups. Statistics were obtained with a two-tailed Student's t test using Graphpad prism.

Molecular structures overlap of PP and Niclo

The FLAP software (vers.2.0), based on the GRID MIFs (Molecular Interaction Fields), served to overlap PP and Niclo molecular structures, in order to find the best 3D molecular alignment according to pharmacophoric descriptors, encoding the essential molecular interactions that small molecules can have. FLAP allows 3D molecular superimposition of one drug with another one and computes a pairwise similarity scores (structural 3D pharmacophore descriptors). Through the FLAP algorithm, four-point pharmacophores are derived from molecular interaction fields (MIFs) based on the three GRID interaction probes and subsequently those 4-point pharmacophores are used to align molecules. PP and Niclos structures were loaded in the FLAP database, as .sdf ASCII files, instructed to generate a maximum of 50 conformers with RMSD value between two conformers of 0.3 Å and an energy window of 20 kcal·mol-1 maximum. Molecular interaction fields (MIFs) were computed with the ligands at specific grid points, by using the H (shape), DRY (hydrophobic),N1 (H-bond acceptor) and O (H-bond donor) probes, as defined in the GRID force field (*66*) with a grid spatial resolution of 0.75 Å. After the overlapping step, the global score (GLOB-S) was obtained as the sum of the similarity scores of each GRID probes. The GLOB-S score ranges from 0 (no similarity) to 1 (maximum similarity). We selected the GLOB-S score because was more suitable to detect global similarity by taking into account the sum of the main hydrophobic, polar and shape overlapping. In addition, 2D-topological molecular fingerprints (MACCS keys) were also calculated for both PP and Niclo and compared each other by using the Tanimoto similarity coefficient, with the aim to confirm the high structural distance of those two drugs.

References to supplementary materials and methods

Caldarola S, Amaldi F, Proud CG and Loreni F. Translational regulation of terminal oligopyrimidine mRNAs induced by serum and amino acids involves distinct signaling events. J Biol Chem. 2004; 279(14):13522-13531.
