# Supplementary material for: Computational drugs repositioning identifies inhibitors of oncogenic PI3K/AKT/P70S6K-dependent pathways among FDA-approved compounds
Source: Oncotarget. 2016 Aug 16;7(37):58743–58. doi: 10.18632/oncotarget.11318 (PMC5312272; doi:10.18632/oncotarget.11318)
Supplement: Supplementary file 2 [file oncotarget-07-58743-s002.docx]

Table S1. Rank-ordered list of compounds after MANTRA 2.0 query and post-processed selection. List of drugs with significant distances from each node. Drugs common to two or all three networks are indicated.

| **PI3K_Ex9_R (Node #1)** | **PI3K_Ex20_R (Node #2)** | **PI3K_Ex20 inh_F (Node_#3)** |  | **match 1∩2** |  | **Selected drugs (1∩2∩3)** |
| --- | --- | --- | --- | --- | --- | --- |
| trifluoperazine | puromycin | quinostatin |  | 5707885 |  | 5707885 |
| metergoline | quinostatin | trifluoperazine |  | alexidine |  | alexidine |
| quinostatin | benzamil | sirolimus |  | anisomycin |  | anisomycin |
| astemizole | sirolimus | benzamil |  | benzamil |  | benzamil |
| loperamide | anisomycin | bromocriptine |  | benzethonium_chloride |  | benzethonium_chloride |
| perphenazine | emetine | alexidine |  | bromocriptine |  | bromocriptine |
| terfenadine | geldanamycin | niclosamide |  | cicloheximide |  | cicloheximide |
| calmidazolium | etoposide | methylbenzethonium_chloride |  | etacrynic_acid |  | etacrynic_acid |
| wortmannin | alexidine | wortmannin |  | etoposide |  | etoposide |
| 0297417-0002B | cicloheximide | benzethonium_chloride |  | fendiline |  | fendiline |
| alexidine | alvespimycin | puromycin |  | geldanamycin |  | geldanamycin |
| bromocriptine | triamterene | etacrynic_acid |  | isotretinoin |  | isotretinoin |
| methylbenzethonium_chloride | niclosamide | PI3K_Ex9_R |  | LY-294002 |  | LY-294002 |
| sirolimus | etacrynic_acid | methylergometrine |  | mefloquine |  | mefloquine |
| etacrynic_acid | LY-294002 | loperamide |  | mepacrine |  | mepacrine |
| mefloquine | wortmannin | geldanamycin |  | metergoline |  | metergoline |
| syrosingopine | benzethonium_chloride | gossypol |  | methylbenzethonium_chloride |  | methylbenzethonium_chloride |
| chlorprothixene | mepacrine | metergoline |  | niclosamide |  | niclosamide |
| geldanamycin | valinomycin | perphenazine |  | pergolide |  | pergolide |
| benzamil | mefloquine | pimozide |  | prochlorperazine |  | prochlorperazine |
| maprotiline | MG-262 | pyrvinium |  | pyrvinium |  | pyrvinium |
| mebendazole | cephaeline | 5114445 |  | quinostatin |  | quinostatin |
| fluspirilene | trifluoperazine | terfenadine |  | raloxifene |  | raloxifene |
| haloperidol | CP-690334-01 | ivermectin |  | sirolimus |  | sirolimus |
| gossypol | methylbenzethonium_chloride | rifabutin |  | trifluoperazine |  | trifluoperazine |
| niclosamide | monorden | syrosingopine |  | valinomycin |  | valinomycin |
| pimozide | (-)-catechin | 0297417-0002B |  | wortmannin |  | wortmannin |
| prenylamine | 5707885 | mefloquine |  | thapsigargin |  |  |
| raloxifene | AG-028671 | phenazopyridine |  | tretinoin |  |  |
| thapsigargin | raloxifene | prochlorperazine |  |  |  |  |
| homochlorcyclizine | disulfiram | fendiline |  |  |  |  |
| co-dergocrine_mesilate | dinoprost | etoposide |  |  |  |  |
| thioridazine | isotretinoin | calmidazolium |  |  |  |  |
| desipramine | prochlorperazine | lanatoside_C |  |  |  |  |
| fluphenazine | 5224221 | latamoxef |  |  |  |  |
| ionomycin | bromocriptine | mepacrine |  |  |  |  |
| levomepromazine | sodium_phenylbutyrate | HC_toxin |  |  |  |  |
| ciclosporin | fendiline | clomifene |  |  |  |  |
| nortriptyline | tretinoin | cytochalasin_B |  |  |  |  |
| monensin | 12,13-EODE | dequalinium_chloride |  |  |  |  |
| benzethonium_chloride | pergolide | digitoxigenin |  |  |  |  |
| chlorcyclizine | metergoline | apigenin |  |  |  |  |
| ivermectin | thapsigargin | scriptaid |  |  |  |  |
| perhexiline | dihydroergocristine | thioproperazine |  |  |  |  |
| methylergometrine | pyrvinium | antimycin_A |  |  |  |  |
| metitepine |  | valinomycin |  |  |  |  |
| suloctidil |  | dexverapamil |  |  |  |  |
| tetrandrine |  | co-dergocrine_mesilate |  |  |  |  |
|  |  |  |  |  |  |  |
| BW-B70C |  | disulfiram |  |  |  |  |
| clofazimine |  | homochlorcyclizine |  |  |  |  |
| pyrvinium |  | ouabain |  |  |  |  |
| pergolide |  | digoxin |  |  |  |  |
| fendiline |  | triamterene |  |  |  |  |
| mepacrine |  | tyloxapol |  |  |  |  |
| podophyllotoxin |  | 5707885 |  |  |  |  |
| tonzonium_bromide |  | CP-320650-01 |  |  |  |  |
| lanatoside_C |  | anisomycin |  |  |  |  |
| prochlorperazine |  | fluphenazine |  |  |  |  |
| mometasone |  | suloctidil |  |  |  |  |
| rottlerin |  | zardaverine |  |  |  |  |
| colforsin |  | LY-294002 |  |  |  |  |
| digoxigenin |  | Prestwick-559 |  |  |  |  |
| CP-645525-01 |  | piperacetazine |  |  |  |  |
| pararosaniline |  | tonzonium_bromide |  |  |  |  |
| tretinoin |  | helveticoside |  |  |  |  |
| 0175029-0000 |  | vorinostat |  |  |  |  |
| proscillaridin |  | tamoxifen |  |  |  |  |
| cytochalasin_B |  | chlorzoxazone |  |  |  |  |
| econazole |  | meptazinol |  |  |  |  |
| cloperastine |  | AG-013608 |  |  |  |  |
| ouabain |  | perhexiline |  |  |  |  |
| albendazole |  | clofilium_tosylate |  |  |  |  |
| piperacetazine |  | maprotiline |  |  |  |  |
| hydroflumethiazide |  | bepridil |  |  |  |  |
| oxetacaine |  | clindamycin |  |  |  |  |
| terbutaline |  | cloperastine |  |  |  |  |
| digoxin |  | idoxuridine |  |  |  |  |
| helveticoside |  | mebendazole |  |  |  |  |
| tamoxifen |  | 1,5-isoquinolinediol |  |  |  |  |
| dipivefrine |  | chlorprothixene |  |  |  |  |
| tyrphostin_AG-1478 |  | alpha-ergocryptine |  |  |  |  |
| clomipramine |  | MS-275 |  |  |  |  |
| clemastine |  | hydroflumethiazide |  |  |  |  |
| cyproheptadine |  | tyrphostin_AG-1478 |  |  |  |  |
| amitriptyline |  | 0175029-0000 |  |  |  |  |
| bepridil |  | emetine |  |  |  |  |
| metixene |  | levomepromazine |  |  |  |  |
| protriptyline |  | diltiazem |  |  |  |  |
| thiostrepton |  | metitepine |  |  |  |  |
| chlorpromazine |  | prenylamine |  |  |  |  |
| thioproperazine |  | rottlerin |  |  |  |  |
| corticosterone |  | naftopidil |  |  |  |  |
| etoposide |  | norcyclobenzaprine |  |  |  |  |
| anisomycin |  | amiodarone |  |  |  |  |
| amoxapine |  | proscillaridin |  |  |  |  |
| 5707885 |  | acepromazine |  |  |  |  |
| dosulepin |  | luteolin |  |  |  |  |
| norcyclobenzaprine |  | pararosaniline |  |  |  |  |
| LY-294002 |  | amoxapine |  |  |  |  |
| lasalocid |  | cefalotin |  |  |  |  |
| latamoxef |  | cicloheximide |  |  |  |  |
| nocodazole |  | diperodon |  |  |  |  |
| Prestwick-559 |  | phenoxybenzamine |  |  |  |  |
| clomifene |  | pronetalol |  |  |  |  |
| isotretinoin |  | thioridazine |  |  |  |  |
| cicloheximide |  | digoxigenin |  |  |  |  |
| naftidrofuryl |  | econazole |  |  |  |  |
| **PI3K_Ex9_R (Node #1)** | **PI3K_Ex20_R (Node #2)** | **PI3K_Ex20 inh_F (Node_#3)** |  | **match 1∩2** |  | **Selected drugs (1∩2∩3)** |
| phenazopyridine |  | 5224221 |  |  |  |  |
| troglitazone |  | berberine |  |  |  |  |
| clozapine |  | flufenamic_acid |  |  |  |  |
| digitoxigenin |  | fluvoxamine |  |  |  |  |
| apigenin |  | metixene |  |  |  |  |
| fenoterol |  | phenelzine |  |  |  |  |
| spiperone |  | mercaptopurine |  |  |  |  |
| zardaverine |  | CP-690334-01 |  |  |  |  |
| clotrimazole |  | desipramine |  |  |  |  |
| colchicine |  | hesperidin |  |  |  |  |
| dihydroergotamine |  | pyrimethamine |  |  |  |  |
| valinomycin |  | raloxifene |  |  |  |  |
| nicergoline |  | heliotrine |  |  |  |  |
| paclitaxel |  | phthalylsulfathiazole |  |  |  |  |
| azathioprine |  | corticosterone |  |  |  |  |
| nimesulide |  | meclozine |  |  |  |  |
| rescinnamine |  | nicergoline |  |  |  |  |
|  |  | dextromethorphan |  |  |  |  |
|  |  | mifepristone |  |  |  |  |
|  |  | amiloride |  |  |  |  |
|  |  | azathioprine |  |  |  |  |
|  |  | corbadrine |  |  |  |  |
|  |  | ketanserin |  |  |  |  |
|  |  | skimmianine |  |  |  |  |
|  |  | zimeldine |  |  |  |  |
|  |  | STOCK1N-35215 |  |  |  |  |
|  |  | cefalexin |  |  |  |  |
|  |  | Prestwick-685 |  |  |  |  |
|  |  | fluspirilene |  |  |  |  |
|  |  | lobeline |  |  |  |  |
|  |  | scopolamine_N-oxide |  |  |  |  |
|  |  | sodium_phenylbutyrate |  |  |  |  |
|  |  | tetrandrine |  |  |  |  |
|  |  | Prestwick-1084 |  |  |  |  |
|  |  | ethoxyquin |  |  |  |  |
|  |  | sulfathiazole |  |  |  |  |
|  |  | acetylsalicylic_acid |  |  |  |  |
|  |  | albendazole |  |  |  |  |
|  |  | glibenclamide |  |  |  |  |
|  |  | trichostatin_A |  |  |  |  |
|  |  | 0317956-0000 |  |  |  |  |
|  |  | 5140203 |  |  |  |  |
|  |  | alvespimycin |  |  |  |  |
|  |  | amprolium |  |  |  |  |
|  |  | cinchocaine |  |  |  |  |
|  |  | doxazosin |  |  |  |  |
|  |  | ellipticine |  |  |  |  |
|  |  | exemestane |  |  |  |  |
|  |  | pargyline |  |  |  |  |
|  |  | pergolide |  |  |  |  |
|  |  | quinisocaine |  |  |  |  |
|  |  | BW-B70C |  |  |  |  |
|  |  | azacitidine |  |  |  |  |
|  |  | clomipramine |  |  |  |  |
|  |  | labetalol |  |  |  |  |
|  |  | monorden |  |  |  |  |
|  |  | chlorcyclizine |  |  |  |  |
|  |  | gefitinib |  |  |  |  |
| **PI3K_Ex9_R (Node #1)** | **PI3K_Ex20_R (Node #2)** | **PI3K_Ex20 inh_F (Node_#3)** |  | **match 1∩2** |  | **Selected drugs (1∩2∩3)** |
|  |  | ornidazole |  |  |  |  |
|  |  | promazine |  |  |  |  |
|  |  | tribenoside |  |  |  |  |
|  |  | chloramphenicol |  |  |  |  |
|  |  | AG-028671 |  |  |  |  |
|  |  | CP-645525-01 |  |  |  |  |
|  |  | MG-262 |  |  |  |  |
|  |  | fulvestrant |  |  |  |  |
|  |  | phenazone |  |  |  |  |
|  |  | alfuzosin |  |  |  |  |
|  |  | bromperidol |  |  |  |  |
|  |  | diazoxide |  |  |  |  |
|  |  | dilazep |  |  |  |  |
|  |  | procainamide |  |  |  |  |
|  |  | protriptyline |  |  |  |  |
|  |  | betazole |  |  |  |  |
|  |  | chlorpromazine |  |  |  |  |
|  |  | clonidine |  |  |  |  |
|  |  | proguanil |  |  |  |  |
|  |  | sulfadiazine |  |  |  |  |
|  |  | practolol |  |  |  |  |
|  |  | betaxolol |  |  |  |  |
|  |  | colforsin |  |  |  |  |
|  |  | solanine |  |  |  |  |
|  |  | bisacodyl |  |  |  |  |
|  |  | doxylamine |  |  |  |  |
|  |  | famprofazone |  |  |  |  |
|  |  | hyoscyamine |  |  |  |  |
|  |  | sulconazole |  |  |  |  |
|  |  | propylthiouracil |  |  |  |  |
|  |  | hydrastine_hydrochloride |  |  |  |  |
|  |  | oxaprozin |  |  |  |  |
|  |  | 1,4-chrysenequinone |  |  |  |  |
|  |  | abamectin |  |  |  |  |
|  |  | liothyronine |  |  |  |  |
|  |  | propafenone |  |  |  |  |
|  |  | isoxsuprine |  |  |  |  |
|  |  | pivampicillin |  |  |  |  |
|  |  | STOCK1N-28457 |  |  |  |  |
|  |  | betahistine |  |  |  |  |
|  |  | dihydroergotamine |  |  |  |  |
|  |  | levcycloserine |  |  |  |  |
|  |  | astemizole |  |  |  |  |
|  |  | dinoprost |  |  |  |  |
|  |  | doxorubicin |  |  |  |  |
|  |  | sulfamethoxypyridazine |  |  |  |  |
|  |  | 0179445-0000 |  |  |  |  |
|  |  | SC-58125 |  |  |  |  |
|  |  | amikacin |  |  |  |  |
|  |  | demeclocycline |  |  |  |  |
|  |  | dimethyloxalylglycine |  |  |  |  |
|  |  | ebselen |  |  |  |  |
|  |  | isotretinoin |  |  |  |  |
|  |  | raubasine |  |  |  |  |
|  |  | troglitazone |  |  |  |  |
|  |  | Prestwick-860 |  |  |  |  |
|  |  | progesterone |  |  |  |  |
|  |  | terbutaline |  |  |  |  |
| **PI3K_Ex9_R (Node #1)** | **PI3K_Ex20_R (Node #2)** | **PI3K_Ex20 inh_F (Node_#3)** |  | **match 1∩2** |  | **Selected drugs (1∩2∩3)** |
|  |  | trimipramine |  |  |  |  |
|  |  | valproic_acid |  |  |  |  |
|  |  | desoxycortone |  |  |  |  |
|  |  | fenoprofen |  |  |  |  |
|  |  | mesalazine |  |  |  |  |
|  |  | (-)-MK-801 |  |  |  |  |
|  |  | cefotaxime |  |  |  |  |
|  |  | cephaeline |  |  |  |  |
|  |  | metamizole_sodium |  |  |  |  |
|  |  | tranylcypromine |  |  |  |  |
|  |  | glafenine |  |  |  |  |
|  |  | hydrastinine |  |  |  |  |
|  |  | iobenguane |  |  |  |  |
|  |  | metyrapone |  |  |  |  |
|  |  | bisoprolol |  |  |  |  |
|  |  | gliclazide |  |  |  |  |
|  |  | iopanoic_acid |  |  |  |  |
|  |  | nortriptyline |  |  |  |  |
|  |  | 0225151-0000 |  |  |  |  |
|  |  | PHA-00665752 |  |  |  |  |
|  |  | clorgiline |  |  |  |  |
|  |  | dirithromycin |  |  |  |  |
|  |  | fenoterol |  |  |  |  |
|  |  | imidurea |  |  |  |  |
|  |  | promethazine |  |  |  |  |
|  |  | salsolidin |  |  |  |  |
|  |  | isoetarine |  |  |  |  |
|  |  | ticlopidine |  |  |  |  |
|  |  | levamisole |  |  |  |  |
|  |  | miconazole |  |  |  |  |
|  |  | monobenzone |  |  |  |  |
|  |  | withaferin_A |  |  |  |  |
|  |  | bufexamac |  |  |  |  |
|  |  | celastrol |  |  |  |  |
|  |  | clofazimine |  |  |  |  |
|  |  | diclofenac |  |  |  |  |
|  |  | estriol |  |  |  |  |
|  |  | kinetin |  |  |  |  |
|  |  | novobiocin |  |  |  |  |
|  |  | piribedil |  |  |  |  |
|  |  | piroxicam |  |  |  |  |
|  |  | amoxicillin |  |  |  |  |
|  |  | blebbistatin |  |  |  |  |
|  |  | gramine |  |  |  |  |
|  |  | pyridoxine |  |  |  |  |
|  |  | sulfaphenazole |  |  |  |  |
|  |  | 4,5-dianilinophthalimide |  |  |  |  |
|  |  | azacyclonol |  |  |  |  |
|  |  | carbachol |  |  |  |  |
|  |  | gallamine_triethiodide |  |  |  |  |
|  |  | lactobionic_acid |  |  |  |  |
|  |  | procarbazine |  |  |  |  |
|  |  | dosulepin |  |  |  |  |
|  |  | etidronic_acid |  |  |  |  |
|  |  | pentetrazol |  |  |  |  |
|  |  | proxyphylline |  |  |  |  |
|  |  | rofecoxib |  |  |  |  |
|  |  | L-methionine_sulfoximine |  |  |  |  |
| **PI3K_Ex9_R (Node #1)** | **PI3K_Ex20_R (Node #2)** | **PI3K_Ex20 inh_F (Node_#3)** |  | **match 1∩2** |  | **Selected drugs (1∩2∩3)** |
|  |  | butoconazole |  |  |  |  |
|  |  | dipivefrine |  |  |  |  |
|  |  | trioxysalen |  |  |  |  |
|  |  | Prestwick-665 |  |  |  |  |
|  |  | chlorphenesin |  |  |  |  |
|  |  | denatonium_benzoate |  |  |  |  |
|  |  | palmatine |  |  |  |  |
|  |  | salbutamol |  |  |  |  |
|  |  | xylazine |  |  |  |  |
|  |  | altizide |  |  |  |  |
|  |  | budesonide |  |  |  |  |
|  |  | carmustine |  |  |  |  |
|  |  | enoxacin |  |  |  |  |
|  |  | menadione |  |  |  |  |
|  |  | dexibuprofen |  |  |  |  |
|  |  | doxepin |  |  |  |  |
|  |  | ethaverine |  |  |  |  |
|  |  | eucatropine |  |  |  |  |
|  |  | flunixin |  |  |  |  |
|  |  | tiapride |  |  |  |  |
|  |  | etofenamate |  |  |  |  |

**Table S2. Drugs Communities enrichments of networks 1, 2 or 3**. Enrichment was significant for pValue *<0,05*. Drugs classes enriched and common to all three networks are indicated in bold.

|  | **Community** | **Drug class** | **pValue** |
| --- | --- | --- | --- |
| **Network 3** | **100** | Antipsychotics (Phenothiazines) | **1,33E-76** |
|  | **62** | Sodium/Calcium Decreasers and calcium channel blockers | **1,51E-22** |
|  | 90 |  | 2,91E-13 |
|  | **16** | PI3K inhibitors | **9,61E-13** |
|  | 32 |  | 3,67E-12 |
|  | **63** | Na+/K+ - ATPase (sodium Potassium) membrane pump inhibitors | **8,70E-10** |
|  | 73 |  | 5,32E-09 |
|  | **34** | antihistamines and anticholinergics | **7,63E-09** |
|  | 52 |  | 7,71E-09 |
|  | **40** |  | **5,02E-08** |
|  | 31 |  | 1,05E-07 |
|  | 42 |  | 7,81E-07 |
|  | 13 |  | 3,61E-06 |
|  | 4 |  | 7,99E-06 |
|  | **28** | HSP90 inhibitors | **1,36E-05** |
|  | 60 |  | 3,62E-05 |
|  | **53** | anisomycin,emetine,cicloheximide,cephaeline | **3,94E-05** |
|  | 49 |  | 6,91E-05 |
|  | 11 |  | 8,91E-05 |
|  | 14 |  | 0,000237657 |
|  | 89 |  | 0,000287085 |
|  | 106 |  | 0,00030114 |
|  | 104 |  | 0,000459028 |
|  | 61 |  | 0,001043791 |
|  | 1 |  | 0,001284689 |
|  | 46 |  | 0,002181364 |
|  | 19 |  | 0,003411467 |
|  |  |  |  |
|  |  |  |  |
| **Network 1** | 100 | Antipsychotics (Phenothiazines) | 1,12E-67 |
|  | 34 | antihistamines and anticholinergics | 2,52E-10 |
|  | 63 | Na+/K+ - ATPase (sodium Potassium) membrane pump inhibitors | 4,57E-08 |
|  | 62 | Sodium/Calcium Decreasers and calcium channel blockers | 1,18E-07 |
|  | 40 |  | 5,66E-05 |
|  |  |  |  |
|  |  |  |  |
| **Network 2** | 53 | anisomycin,emetine,cicloheximide,cephaeline | 3,94E-05 |
|  | 40 |  | 0,000423139 |
|  | 28 | HSP90 inhibitors | 0,000662057 |
|  | 100 | Antipsychotics (Phenothiazines) | 0,003347342 |
|  | 62 | Sodium/Calcium Decreasers and calcium channel blockers | 0,006950532 |
|  | 16 | PI3K inhibitors | 0,026657155 |
|  |  |  |  |

**Table S3. Short list of genes from HME PIK3CA(E545K)-reverse signature predicted being regulated by Niclo or PP** (see Materials and Methods): genes are annotated according HG-U133A Affymetrix array probes ID.

| **Upregulated genes** |
| --- |
| *hCCNG2 -* 202769_at |
| *hHBP1 -* 209102_s_at |
| *hHEY1 -* 218839_at |
| *hIL8 -* 202859_x_at |
| *hCSGALNACT1_*219049_at |
| *hTGFA -*205015-s-at |
|  |
| **Downregulated genes** |
| ***hCCNE2* - 205034_at** |
| ***hDSCC1* - 219000_s_at** |
| ***hENDOD1* - 212573_at** |
| ***hPPP2R1B* - 202883_s_at** |
|  |
